# Antigenic Fingerprinting of H5N1 Avian Influenza Using Convalescent Sera and Monoclonal Antibodies Reveals Potential Vaccine and Diagnostic Targets

**DOI:** 10.1371/journal.pmed.1000049

**Published:** 2009-04-21

**Authors:** Surender Khurana, Amorsolo L. Suguitan, Yonaira Rivera, Cameron P. Simmons, Antonio Lanzavecchia, Federica Sallusto, Jody Manischewitz, Lisa R. King, Kanta Subbarao, Hana Golding

**Affiliations:** 1 Division of Viral Products, Center for Biologics Evaluation and Research (CBER), Food and Drug Administration, Bethesda, Maryland, United States of America; 2 Laboratory of Infectious Diseases, National Institute of Allergy and Infectious Diseases, National Institutes of Health, Bethesda, Maryland, United States of America; 3 Oxford University Clinical Research Unit, Hospital for Tropical Diseases, Ho Chi Minh City, Vietnam; 4 Institute for Research in Biomedicine, Bellinzona, Switzerland; The University of Hong Kong, Hong Kong

## Abstract

**Background:**

Transmission of highly pathogenic avian H5N1 viruses from poultry to humans have raised fears of an impending influenza pandemic. Concerted efforts are underway to prepare effective vaccines and therapies including polyclonal or monoclonal antibodies against H5N1. Current efforts are hampered by the paucity of information on protective immune responses against avian influenza. Characterizing the B cell responses in convalescent individuals could help in the design of future vaccines and therapeutics.

**Methods and Findings:**

To address this need, we generated whole-genome–fragment phage display libraries (GFPDL) expressing fragments of 15–350 amino acids covering all the proteins of A/Vietnam/1203/2004 (H5N1). These GFPDL were used to analyze neutralizing human monoclonal antibodies and sera of five individuals who had recovered from H5N1 infection. This approach led to the mapping of two broadly neutralizing human monoclonal antibodies with conformation-dependent epitopes. In H5N1 convalescent sera, we have identified several potentially protective H5N1-specific human antibody epitopes in H5 HA[(-10)-223], neuraminidase catalytic site, and M2 ectodomain. In addition, for the first time to our knowledge in humans, we identified strong reactivity against PB1-F2, a putative virulence factor, following H5N1 infection. Importantly, novel epitopes were identified, which were recognized by H5N1-convalescent sera but did not react with sera from control individuals (H5N1 naïve, H1N1 or H3N2 seropositive).

**Conclusion:**

This is the first study, to our knowledge, describing the complete antibody repertoire following H5N1 infection. Collectively, these data will contribute to rational vaccine design and new H5N1-specific serodiagnostic surveillance tools.

## Abstract 2

Using whole-genome-fragment phage display libraries, Hana Golding and colleagues identify the viral epitopes recognized by serum antibodies in humans who have recovered from infection with H5N1 avian influenza.

## Editors' Summary

**Background**

Every winter, millions of people catch influenza, a viral infection of the airways. Most recover quickly but seasonal influenza outbreaks (epidemics) kill about half a million people annually. These epidemics occur because small but frequent changes in the viral proteins (antigens) to which the human immune system responds mean that an immune response produced one year by infection or through vaccination provides only partial protection against influenza the next year. Influenza viruses also occasionally appear that contain major antigenic changes. Human populations have little or no immunity to such viruses (which often originate in animals or birds), so they can start deadly global epidemics (pandemics ). Worryingly, the last influenza pandemic occurred in 1968 and many experts fear that another pandemic is now overdue. The trigger for such a pandemic, they think, could be the avian (bird) H5N1 influenza virus, which first appeared in 1996 in a goose in China. The name indicates the types of two major influenza antigens present in the virus: H5N1 carries type 5 hemagglutinin and type 1 neuraminidase.

**Why Was This Study Done?**

H5N1 has caused about 400 confirmed cases of human influenza and more than 250 deaths in the past decade but it has not started a human pandemic because it cannot pass easily between people. However, it could possibly acquire this ability at any time, so it is a priority to develop both vaccines that will provide protection against a pandemic H5N1 viral strain, as well as antibody-based antiviral therapies for people not protected by vaccination (antibodies are proteins produced by the immune system that help to fight infections; people can sometimes be protected from infection by injecting them with pre-prepared antibodies). To do this, scientists need to know how the human immune system responds to the H5N1 virus. In particular, they need to know which parts of the virus the immune system can detect and make antibodies against. In this study, therefore, the researchers characterize the specific antibody responses found in people recovering from infection with H5N1.

**What Did the Researchers Do and Find?**

The researchers made several “genome-fragment phage display libraries”, collections of bacterial viruses (phages) engineered so that each phage makes one of many possible short pieces (polypeptides) of a nonphage protein. Such “libraries” can be used to investigate which fragments are recognized by antibodies from a given source. In this case, several libraries were made that contained fragments of the genome of the H5N1 strain responsible for an outbreak of human influenza in Vietnam in 2004–2005 (A/Vietnam/1203/2004). The researchers used these libraries to analyze the antibodies made by five Vietnamese people recovering from infection with A/Vietnam/1203/2004. H5N1 convalescent blood samples, the researchers report, contained antibodies that recognized small regions (“epitopes”) in several viral proteins, including hemagglutinin, neuraminidase, a structural protein called M2, and a viral protein called PB1-F2 that is partly responsible for the severity of H5N1 infections. Several of the novel epitopes identified were not recognized by antibodies in blood taken from people recovering from infection with other influenza viruses. The researchers also used their phage display libraries to analyze two neutralizing human monoclonal antibodies generated from patients infected with A/Vietnam/1203/2004 (neutralizing antibodies protect mice against normally lethal challenge with H5N1; monoclonal antibodies are generated in the laboratory by creating continuously growing cell lines that produce a single type of antibody). Importantly, both of the neutralizing monoclonal antibodies recognized “noncontinuous conformation-dependent epitopes”—protein sequences that are not adjacent to one another in the polypeptide sequence of the protein, but that lie close together in space because of the way the protein is folded up.

**What Do These Findings Mean?**

Although some aspects of the antibody repertoire produced in people exposed to the H5N1 influenza virus may have been missed in this analysis, these findings provide important and detailed new information about how the human immune system responds to infection with this virus. In particular, they show that people recovering from H5N1 infection make a diverse range of antibodies against several viral proteins for at least six months and identify specific parts of H5N1 that may be particularly good at stimulating a protective immune response. This information can now be used to help design vaccines against H5N1 and antibody-based therapies for the treatment of H5N1 infections, and to develop new tools for monitoring outbreaks of avian influenza in human populations.

**Additional Information**

Please access these Web sites via the online version of this summary at http://dx.doi.org/10.1371/journal.pmed.1000049.

- This study is further discussed in a *PLoS Medicine* Perspective by Malik Peiris
- The US Centers for Disease Control and Prevention provides information for about influenza for patients and professionals, including specific information on avian and pandemic influenza (in several languages)
- The World Health Organization provides information on influenza (in several languages) and on H5N1 avian influenza (in several languages), and a global timeline about H5N1 avian influenza infection in birds and people
- The UK Health Protection Agency provides information on avian, pandemic, and epidemic (seasonal) influenza
- MedlinePlus provides a list of links to other information about influenza and bird flu (in English and Spanish)

## Introduction

The recent spread of highly pathogenic (HP) H5N1 avian influenza viruses (AIV) among poultry and transmission of these viruses to humans raised concerns of a potential influenza pandemic. In preparation for such an event, world-wide efforts are under way to test and stockpile preventive vaccines, antiviral drugs, and passive immune therapies [1], [2].

Such efforts could be greatly enhanced by understanding the immune responses of individuals who survived H5N1 virus infection. Recently, human monoclonal antibodies (MAbs) were generated from patients in Vietnam and Turkey who had recovered from H5N1 infections [3], [4]. Some of the human MAbs demonstrated cross-clade neutralization in vitro and protected mice from challenge with lethal H5N1 viruses. The potential benefit of antibody therapy was suggested during the Spanish influenza outbreak when transfusion of convalescent sera reduced mortality by >50% [5], and in a patient infected with an H5N1 (clade 2.3) virus in China [6].

However, there are significant gaps in our knowledge of antibody epitopes against H5N1 viruses, especially in humans, and only a few epitopes have been identified in proteins other than the haemagglutinin (HA) [7]. Characterizing the B cell responses in convalescent individuals could help in the design of future vaccines and therapeutics. Finally, identification of long-lasting antibodies against conserved epitopes could assist in the development of serological assays for surveillance of AIV infections.

To address these gaps we have constructed whole-genome–fragment phage display libraries (GFPDL) expressing all the open reading frames of H5N1 A/Vietnam/1203/2004. The H5N1 GFPDL were used to identify recognition sites of antibodies in convalescent sera obtained from five Vietnamese individuals with a history of H5N1 infection and two H5-specific neutralizing MAbs derived from two of these survivors.

## Materials and Methods

### Plasma Samples and MAbs

Serum samples from five patients who survived H5N1 infection in Vietnam were obtained at one time point within 2–6 mo following H5N1 infection in 2004 and were previously described (Viet-1, 24-year-old male; Viet-2, 8-year-old female; Viet-3 25-year-old female; Viet-4, 26-year-old female; Viet-5, 23-year-old male) [8]. MAbs FLA5.10, and FLD21.140 were derived from two of these donors and were previously described [3]. Plasma samples from 20 Vietnamese adults (all females, aged 20–39 y) and resident in Vietnam but with no history of H5N1 exposure were used as controls in antibody binding experiments. In microneutralization assays using A/Wisconsin/67/05 (H3N2), A/New York/55/04 (H3N2), A/New Caledonia/22/99 (H1N1), and A/Solomon Islands/03/06 (H1N1), 75% of these plasma had neutralizing antibodies against either H3N2 strains, H1N1 strains, or both, with titers ranging between 1∶40 to 1∶1,280. Ten plasma samples from US residents with culture confirmed human seasonal influenza infections who had high HI titers against both H1N1 and H3N2 strains circulation during the 2004–2007 seasons were used as additional controls (no possible H5N1 exposure). All samples were de-identified. All protocols were evaluated by the CBER/NIH Research Involving Human Subjects Committee and were conducted under RIHSC exemption number 03-118B.

### Construction of H5N1 Gene-Fragmented Phage Display Libraries

cDNA corresponding to all eight gene segments of the A/H5N1/Vietnam/1203/2004 were generated from RNA isolated from egg-grown virus strain, and were used for cloning. fSK-9-3 is a gIIIp display-based phage vector where the desired polypeptide can be expressed as gIIIp fusion protein.

Phage display libraries were constructed individually for HA and neuraminidase (NA) genes (referred to as HA-NA) and the rest of the six gene segments (PB2, PB1, PA, NP, M, and NS), referred to as FLU-6. Purified DNA containing equimolar ratio of HA and NA (HA-NA) or of the six genes (FLU-6) were digested with DNase shotgun cleavage kit (Novagen) per manufacturer's instructions, to obtain DNA fragments in the size range of 50–200 and 200–1,000 bp for each of the two gene segment pools. Detailed methodology for library construction was described previously [9].

Four libraries were constructed: fSK9-3 H5Viet-HA-NA (50–200 bp), fSK9-3 H5Viet-HA-NA (200–1,000 bp), fSK9-3 H5Viet-FLU-6 (50–200 bp), and fSK9-3 H5Viet-FLU-6 (200–1,000 bp) (Figure S2).

### Random Peptide Library

A random linear dodecapeptide phage display library (Ph.D-12), wherein the displayed peptides (12-mer) are expressed fused to the N terminus of gIII protein was purchased from New England Biolabs.

### Affinity Selection of H5N1 GFPDL Phages with MAb or Polyclonal Human Sera

Prior to panning of GFPDL with plate-bound polyclonal serum antibodies, serum components, which could nonspecifically interact with phage proteins, were removed by incubation with UV-killed M13K07 phage-coated Petri dishes. Subsequent GFPDL affinity selection was carried out on antibody-coated wells as well as in-solution (with Protein A/G).

For in-solution panning, 10^10^ phages (of the Influenza H5 GFPDL) in 500 µl PBST containing 1% BSA were preincubated with 200 µl of 50% Ultralink Protein A/G slurry (Pierce) for 1 h at room temperature (RT) on end-to-end shaker. Following brief centrifugation, 500 µl of supernatant was removed and was added to 5 µg of human anti-H5N1 MAb or 100 µl of VCSM13-preadsorbed human serum (in 1% BSA-PBST), and incubated for 1 h at RT on end-to-end shaker, followed by 200 µl of 50% Ultralink Protein A/G slurry (Pierce) on end-to-end shaker at RT for 1 h. The unbound phages were removed in ten washes with PBST followed by three washes with PBS. The bound phages were eluted by addition of 800 µl of 0.1 N HCl (adjusted to pH 2.2 with glycine and BSA), and incubated for 10 min at RT on end-to-end shaker. The eluates were collected and neutralized by adding 64 µl of 2 M Tris solution. Panning on coated strips has been detailed in [9]. The inserts were PCR amplified and sequenced.

### Peptide ELISA

Biotinylated peptides (1 µg/well) were captured onto wells coated with 500 ng of streptavidin. After blocking with PBST containing 2% milk, serial dilutions of human serum in blocking solution were added to each well, incubated for 1 h at RT, followed by addition of 2,000-fold dilution of HRP-conjugated goat anti-human IgG-Fc specific antibody, and developed by 100 µl of OPD substrate solution. Absorbance was measured at 490–492 nm. As negative controls, peptides derived from HIV and human CCR5 were used.

### Affinity Measurements by Surface Plasmon Resonance

Steady-state equilibrium binding of MAb FLA5.10, and FLD21.140 was monitored at 25°C using a ProteOn surface plasmon resonance biosensor (BioRad Labs). The HA [(-10)-223]-His_6_ was coupled to a GLC sensor chip using amine coupling with 40 resonance units (RU) in the test flow cells. Samples of 60 µl of freshly prepared antibody at various concentrations were injected at a flow rate of 30 µl/min (120-s contact time). Flow was directed over a mock surface to which no protein was bound, followed by the HA [(-10)-223]-His_6_ coupled surface. Responses from the peptide surface were corrected for the response from the mock surface and for responses from a separate, buffer only, injection. MAb 2D7 (anti-CCR5) was used as a negative control antibody in various binding experiments. Binding Kinetics for the MAbs and the data analysis was performed using BioRad ProteON manager software (version 2.0.1). Affinity measurements were calculated using the Langmuir with Mass transfer algorithm.

### Adsorption of Polyclonal Human Sera on H5N1 GFPDL Phages and Residual Reactivity to H5N1-Vietnam HA

Prior to panning of GFPDL, 500 µl of 10-fold diluted pooled serum antibodies from H5N1 survivors were adsorbed by incubation with H5N1 (HA+NA) GFPDL phage-coated Petri dishes. To ascertain the residual antibodies specificity, an ELISA was performed with wells coated with 200 ng/100 µl of recombinant H5 HA (A/Vietnam/1203/2004, Protein Sciences). After blocking with PBST containing 2% milk, serial dilutions of human serum (with or without adsorption) in blocking solution were added to each well, incubated for 1 h at RT, followed by addition of 2,000-fold diluted HRP-conjugated goat anti-human IgG-Fc specific antibody and developed by 100 µl of OPD substrate solution. Absorbance was measured at 490–492 nm.

### Neutralizing Antibodies Adsorption with HA1 peptides

Five-fold diluted immune serum (from sheep or ferret) (500 µl) was added to 0.5 mg of purified HA [(-10)-223]-His_6_ (or shorter HA1-derived peptides) or to control GST-His_6_ protein, and incubated for 1 h at RT. Ni-NTA Sepharose beads (200 µl; Qiagen) were added for 20 min at RT on end-to-end shaker, to capture the His-tagged peptides and the antibodies bound to them, followed by a brief centrifugation. Supernatants containing the unbound antibodies were collected. The pelleted beads were washed five times with PBST, followed by two washes with PBS. Sepharose-peptide–bound antibodies were eluted by incubating beads with 500 µl of 0.1 N HCl (adjusted to [pH 2.2] with glycine and BSA), for 10 min at RT on end-to-end shaker. The eluates were collected and neutralized by adding 40 µl of 2 M Tris solution. In some cases, the serum adsorption was performed using biotinylated peptides, which were captured using streptavidin-coupled magnetic beads.

### Neutralization Assay

Viral-neutralizing activity was analyzed in a microneutralization assay on the basis of the methods of the pandemic influenza reference laboratories of the US Centers for Disease Control (CDC) [10]–[12]. The sheep anti-H5N1 A/Vietnam/1203/2004 (CBER SRID reagent), and sera from ferrets infected with wild-type H5N1 A/Vietnam/1203/2004, were treated with a Receptor Destroying Enzyme (RDE) overnight followed by heat-inactivation. Low pathogenicity H5N1 viruses, generated by reverse genetics, were obtained from St. Jude, CDC, and NIBSC: A/Vietnam/1203/2004 (SJCRH, clade 1), A/Indo/5/2005 (PR8-IBCDC-RG2; clade 2.1), A/turkey/Turkey/1/05 (NIBRG-23; clade 2.2), A/Anhui/1/05 (IBCDC-RG5, clade 2.3.4).

## Results

### Epitope Mapping of H5N1-Neutralizing MAbs

**Figure 1:**
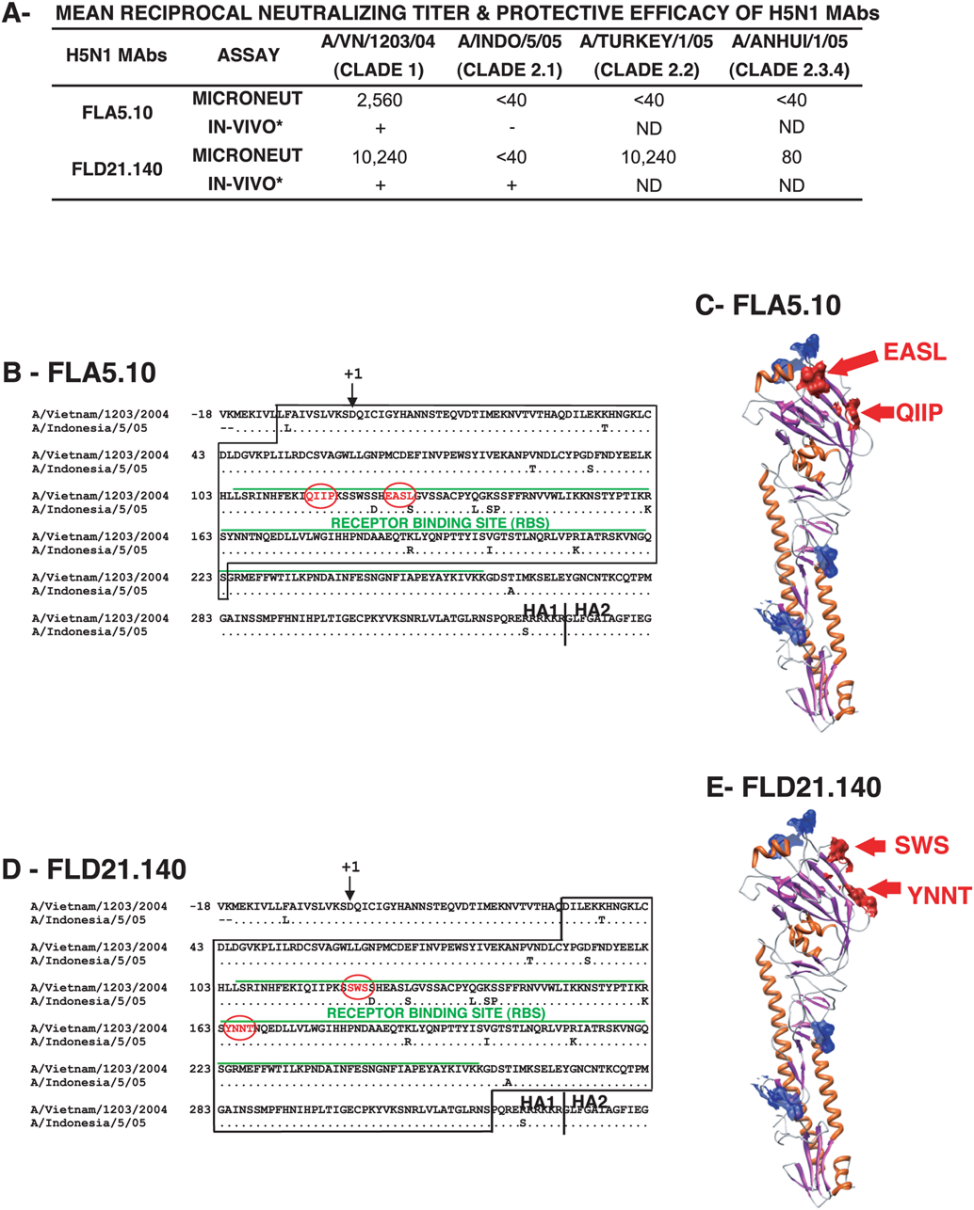
Epitope mapping of broadly neutralizing H5N1 human MAbs. (A) End-point titers (mean of three replicates) using two H5N1 human MAbs (at 1 mg/ml) in a microneutralization assay performed with rgH5N1×PR8 (2∶6) reassorted viruses are shown. *, The information on in vivo protection of mice against wild-type A/Vietnam/1203/2004 and A/Indonesia/5/05 challenge was previously published [3]. (B) HA segment [(-10)-233] was identified by GFPDL panning using MAb FLA5.10 (boxed). Amino acid number +1 corresponds to H3 (A/California/7/2004) amino acid -10. The critical contact residues for FLA5.10, identified using RPL are shown in red circles. (C) Alignment of the critical residues for MAb FLA5.10 binding on the 3-D structure of the HA monomer (Protein Data Base [PDB] identifier 21BX) with amino acid colors corresponding to (B). The predicted glycosylation sites (NXT/NXS) are shown in blue. (D) HA segment (32–320) identified by GFPDL-panning using MAb FLD21.140 (boxed). The putative contact residues identified using RPL, are shown in red circles. (E) Alignment of the critical residues for MAb FLD21.140 binding on the 3-D structure of the HA monomer (PDB Identifier 2IBX) with amino acid colors corresponding to (D). The predicted glycosylation sites (NXT/NXS) are shown in blue.

Following the outbreak of H5N1 AIV in humans in Vietnam (2004–2005), in which 13/18 patients died [8], [13], memory B cells from peripheral blood mononuclear cells of four surviving patients were immortalized with Epstein-Barr virus to generate human MAbs [3]. Two of the human MAbs exhibited distinct patterns of reactivity with clade 1 and clade 2 H5N1 viruses. FLA5.10 had a narrow (clade 1-specific) neutralization range (Figure 1A) and protected mice from lethal challenge with clade 1 but not clade II viruses [3]. In contrast, FLD21.140 demonstrated broader cross-clade neutralization in vitro with very high neutralization titers against A/Vietnam/1203/2004 (clade 1) and A/Turkey/turkey/1/05 (clade 2.2), and a low neutralization titer against A/Anhui/1/05 (clade 2.3.4) (Figure 1A). Interestingly, although this MAb did not neutralize A/Indonesia/5/05 (clade 2.1) in vitro, it protected BALB/c mice from lethal challenge with A/Indonesia/5/05 as previously reported [3]. We predicted that these MAbs have different binding sites, and the epitope of MAb FLD21.140 could be a potential target for cross-reactive H5N1 vaccines.

Epitope mapping was addressed following the construction of GFPDL, spanning the genome of A/Vietnam/1203/2004 (H5N1) (Figure S1). The insert sizes in the separately constructed HA+NA and FLU-6 libraries ranged between 50–200 bp and 200–1,000 bp to allow presentation of conformation-dependent epitopes. The four influenza GFPDL consisted of 9.6×10^6^ to 2.6×10^7^ phages (Figure S2A). PCR analysis of 192 transformants per library confirmed that the size and distribution of inserts was random across all eight genes (Figure S2B).

Using MAb FLA5.10 for panning of the H5N1 HA+NA libraries, multiple phages expressing an HA segment corresponding to amino acids (aa) [(-10)-223] were selected that included part of the receptor binding site (RBS) preceded by an N-terminal sequence (Figure 1B). This HA segment contains 13 aa differences between A/Vietnam/1203/2004 and A/Indonesia/5/05 viruses, any of which could contribute to the clade-restricted binding of this MAb. To delineate the specific contact residues of FLA5.10, we used a random peptide phage display library (RPL), as previously used to map conformation-dependent MAbs [14]. Most of the selected phages displayed a peptide with the consensus sequence [(H/Q)-I-(T/I)-P-X-X-X-E-(A/V)-T-L, where “X” is any amino acid]. This epitope sequence mimics a nonlinear sequence present in the RBS, which aligns with 115-QIIP-118 and 126-EASL-129 (Figure 1B, circled in red). Comparison of this portion of the HA sequence of A/Vietnam/1203/2004 and A/Indonesia/5/05 shows only one amino acid difference (L_129_S). Direct binding of FLA5.10 to a chemically synthesized peptide (5.10-101) and a mutated version of the peptide (5.10-101-L/A) confirmed that L129 is a critical contact residue (Figure S3). Importantly, these aa clusters aligned closely on the outer face of the HA globular head on the 3-D structure of HA and are not expected to be masked by glycosylated residues (shown in blue) (Figure 1C).

GFPDL panning with MAb FLD21.40 identified a large segment (HA 32–320) including the entire RBS. Subsequent panning with RPL narrowed the putative epitope to two aa clusters, 121-SWS-123 and 164-YNNT-167, which were separated by 40 aa in the linear sequence (Figure 1D, circled in red), but were closely located on the 3-D structure of HA monomer (Figure 1E). Importantly, the sequences identified are highly conserved among clade 1 and clade 2 H5N1 viruses (including clades 2.1, 2.2, 2.3.1, 2.3.2, 2.3.3, 2.3.4, 2.4, and 2.5) as well as clades 5, 6, and 8. This conservation may explain the broader cross-neutralization in vitro and cross-protection in vivo conferred by FLD21.140 (Figure 1A).

### Binding of Human MAbs to HA Segment

**Figure 2:**
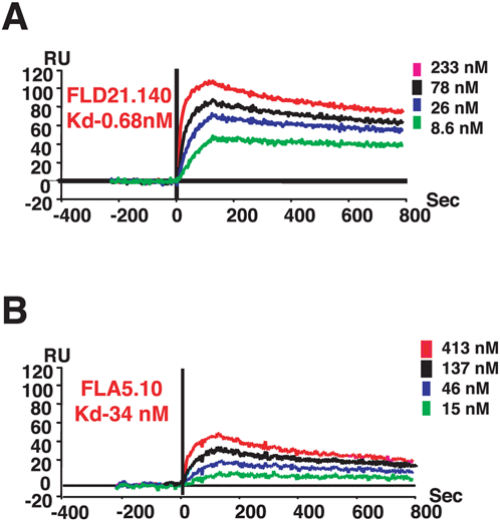
Steady-state binding equilibrium analysis of human MAbs to purified bacterially expressed H5 HA[(-10-223)] fragment. Various concentrations of MAbs FLD21.140 (A) and FLA5.10 (B) were injected simultaneously onto recombinant HA [(-10)-223] (identified in Figure 1B) peptide, immobilized on a sensor chip through the free amine group, and onto a blank flow cell, free of peptide. Binding was recorded using ProteOn system surface plasmon resonance biosensor instrument (BioRad Labs). As a control, anti-CCR5 MAb 2D7) was injected at the same concentrations on HA [(-10)-223] coupled chip. RU, resonance units.

To confirm that the HA segments identified using influenza GFPDL can be recognized by neutralizing human MAbs independent of phage presentation, the H5 HA [(-10)-223] peptide was expressed and purified from *Escherichia coli.* This protein fragment captured on a biosensor chip was used to determine the binding kinetics of FLA5.10 (Figure 2B) and FLD21.140 (Figure 2A). Surprisingly, FLD21.140 bound to this HA segment with 50-fold higher affinity than FLA5.10 (K_d_ of 0.68 nM versus 34 nM, respectively). Such a difference in binding affinities may predict higher avidity of binding to virus in vivo for FLD21.140, and could contribute to its ability to protect mice against clade 2 viruses. Therefore, both specificity and avidity could be factors in heterologous protection.

### Antibody Epitopes in the HA and NA Recognized by Sera from Survivors of H5N1 Infection

The successful use of the GFPDL/RPL in elucidating the conformation-dependent epitopes of two human MAbs provided proof of concept for this approach. Next, it was important to establish if such antibodies are represented in the polyclonal sera of individuals who had recovered from H5N1 virus infection, and to identify other antibody specificities that may have contributed to virus clearance.

Convalescent sera from five H5N1 patients, obtained between 54 and 182 d following hospital admission, were analyzed using the A/Vietnam/1203/2004 GFPDL. We first demonstrated the capacity of the GFPDL to adsorb, at a minimum, 85% of HA-specific antibodies in the pooled convalescent sera, as determined by binding to recombinant H5 HA (A/Vietnam/1203/2004, Protein Sciences) in ELISA (Figure S4), confirming the rationale of using this approach to dissect the antibody repertoires in polyclonal sera.

**Figure 3:**
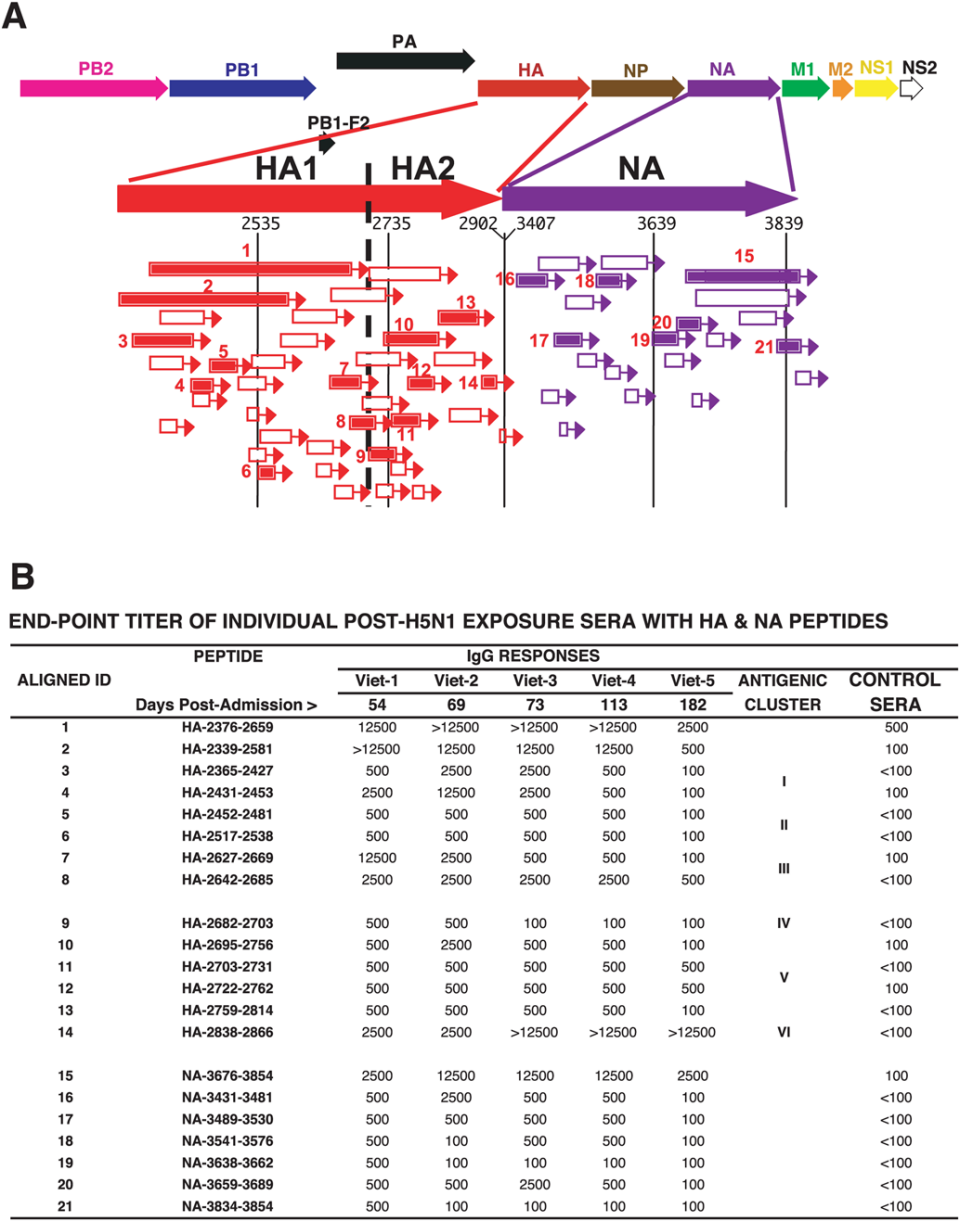
Elucidation of the epitope profile in HA and NA proteins recognized by antibodies in individuals that survived H5N1 infections in Vietnam. (A) Alignment of peptides recognized by pooled sera from H5N1-infected individuals identified using H5 (HA+NA) GFPDL. Bars with arrows represent the identified inserts in 5′–3′ orientation. Numbered segments represent inserts that were selected with high frequencies (≥5; Table S1). These peptides were expressed and purified from *E .coli* or were chemically synthesized and used in ELISA. The sequences of the influenza encoded fragments are numbered according to the intact complete proteome (Figure S1). Peptide ID numbers are the same in (A) and (B). (B) ELISA reactivity of sera from individual H5N1-infected patients (Viet1–5) or pooled sera from 20 healthy Vietnamese adults (75% had neutralizing titers against either H3N2 influenza strains, H1N1 strains, or both) with H5N1 HA1 peptides (1–8), HA2 peptides (9–14), and NA peptides (15–21) (localization indicated in Figure 4A). An initial serum dilution (1∶100) was followed by serial 5-fold dilutions. End-point titers are reported. Days postadmission represent the time of serum collection for each patient. Six antigenic clusters in HA (cluster-I, 2,359–2,453; cluster-II, 2,454–2,621; cluster-III, 2,627–2,670; cluster-IV, 2,682–2,703; cluster-V, 2,706–2,814; cluster-VI, 2,823–2,816) recognized by the convalescent sera are shown.

**Figure 4:**
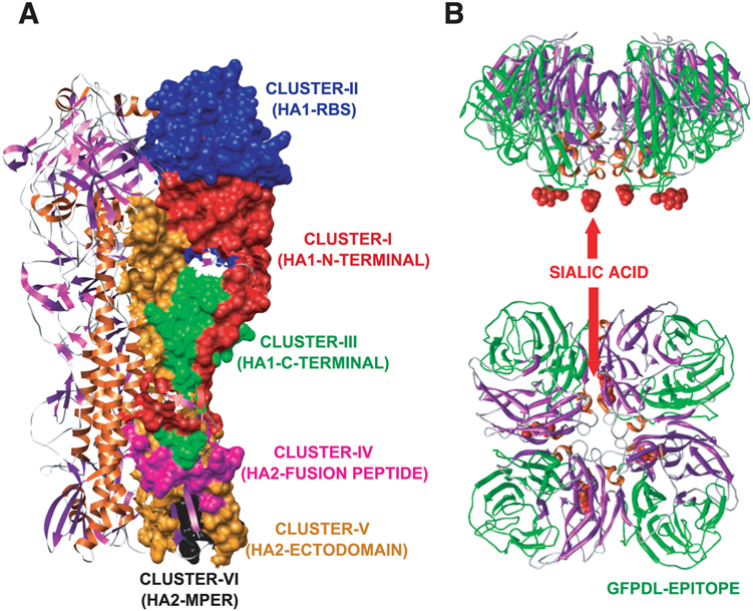
Main antigenic clusters in the structures of HA and NA recognized by antibodies from H5N1 virus infected individuals. (A) Antigenic clusters in HA, as identified in Figure 3B, are shown as surface exposed colored patches on one HA monomer within the HA trimer structure (PDB identifier 2IBX). The antigenic clusters (I–III) in HA1 cover the Antigenic Sites a, b, c, d, and e that have been described in the H3 HA1 based on mouse MAbs [15], [22]. (B) The immunodominant conformational epitope in the NA (NA-3676-3854, peptide 15 in Figure 3B) is shown in green on the tetrameric NA structure (PDB Identifier 2HTY) with the predicted site of bound sialic acid shown in red. Side view and bird-eye views are shown.

Using the HA+NA GFPDL, the pooled convalescent sera recognized a large number of clones in the HA1, HA2, and NA proteins (Figure 3A). The sequences and frequencies of all the peptides that were bound by the pooled sera are presented in Table S1. The most frequently recognized segments (numbered in Figure 3A) were chemically synthesized or expressed in *E. coli* and used in ELISA with individual convalescent sera (Viet 1-5, Figure 3B). All five individual sera reacted with the panel of peptides from HA1, HA2, and NA proteins. The strongest reactivity was observed for the large HA1 peptides 1 and 2 encompassing the RBS, which were also recognized by the human MAbs (unpublished data), and against the shorter peptides 7 and 8 in the C terminus of HA1. Phage clones expressing HA2 sequences were isolated at high frequency (Table S1), and individual binding titers for these peptides were confirmed by ELISA (Figure 3B, peptides 9–14). The predominant HA sites recognized by the convalescent sera could be grouped into six antigenic clusters (I–VI) that mapped to the outer face of the HA trimer (Figures 3B and 4A). The HA antigenic clusters were defined on the basis of sequences of the repeatedly selected phage displayed epitopes (Table S1) and the confirmatory ELISA reactivity profiles with the patients' plasma (Figure 3B). Cluster-I, 2,359–2,453; cluster-II: 2,454–2,621; cluster-III: 2,627–2,670; cluster-IV: 2,682–2,703; cluster-V: 2,706–2,814; cluster-VI: 2,823–2,816. Clusters-I and -II encompass the previously described HA1 antigenic sites “a–e” that were defined primarily using mouse monoclonal antibodies against H3 influenza strains [15], [16].

Although the serum samples were obtained at a single time point postinfection in each patient, a general pattern was observed. The ELISA titers of Viet-5 serum (6 mo postinfection) were lower than for earlier convalescence sera (Figure 3B). Interestingly, high antibody titers against peptide 14 in the HA2 C terminus (HA-2838–2866) were detected in all five samples.

To control for the potentially confounding influence of cross-reactive antibodies elicited by seasonal influenza, we also evaluated the binding profile of sera from 20 healthy Vietnamese adults with no epidemiological exposure to H5N1 (Figure 3B, right column). While modest binding to peptide 1, encompassing most of the HA1 was observed with these sera (titer 1∶500), binding to all the other peptides was very low or absent (≤1∶100). These results were expanded using 10 US individuals with culture confirmed seasonal influenza infections and high HI titers against both H1N1 and H3N2 (Table S2). Student's *t*-test analyses (two-tailed distribution with equal variance and a degree of freedom of 13) revealed that the binding to the H5N1 HA peptides were significantly different (*p*<0.05) between the H5N1 convalescent sera and the plasma from unexposed US and Vietnamese controls (Table S2 and unpublished data). These findings suggested that the antigenic map of the H5 HA revealed in our study is unlikely to have been confounded by the presence of cross-reactive antibodies elicited by exposure to seasonal influenza.

In the NA, a large number of epitopes captured by the pooled convalescent sera were also recognized by the individual sera (Figure 3A and 3B, peptides 15–21). NA peptide 15, which is 178 aa long (NA 269–447), contains the primary residues required for sialic acid binding and the catalytic activity of NA [17] as depicted on the NA structure in Figure 4B. This sequence was recognized at high frequency by the convalescent sera (62 clones, Table S1) and was the only peptide sequence within NA that showed strong reactivity with individual sera in ELISA (Figure 3B). This region is also >85% conserved between the human and AIV N1 NAs of A/New Caledonia/20/99 (H1N1) and A/Vietnam/1203/2004 (H5N1), respectively. Surprisingly, Vietnamese control sera bound peptide 15 minimally (≤1∶100) (Figure 3B), and no reactivity was observed with seasonal influenza infected US control sera (Table S2).

### Antibody Epitopes in the Internal Proteins of Influenza Recognized by Sera from Survivors of H5N1 Infection

**Figure 5:**
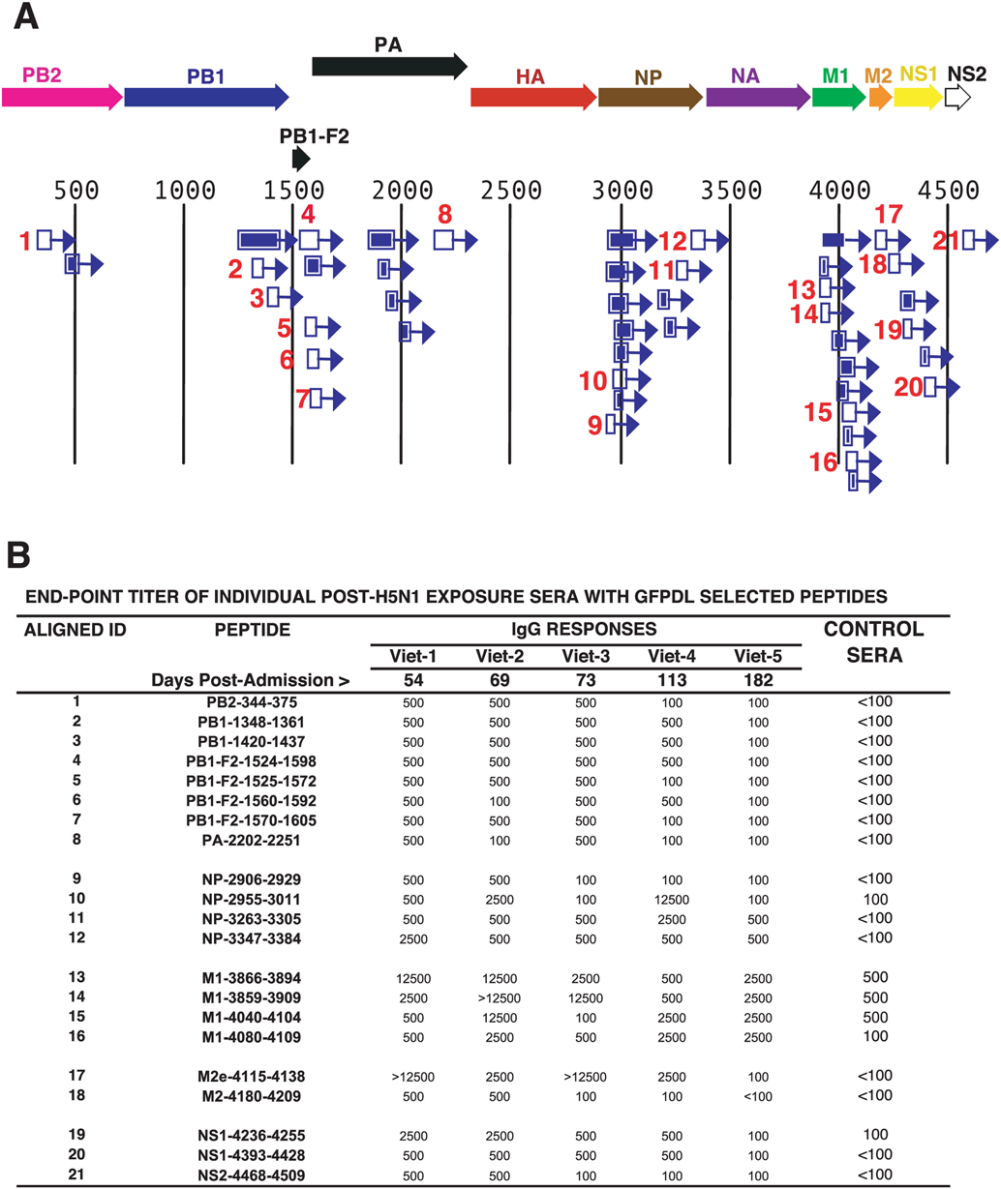
Antibody epitopes in H5N1 internal proteins (FLU-6) recognized by pooled sera from H5N1 infected individuals. (A) Schematic alignment of the peptides identified using GFPDL (H5N1-FLU-6) expressing all internal proteins of influenza A/Vietnam/1203/2004. The predicted influenza encoded proteins are numbered according to the complete proteome (Figure S1). Bars with arrows indicate identified inserts in the 5′–3′ orientation. Numbered segments represent high frequency clones (≥5; Table S1). These peptides were expressed and purified from *E. coli* or were chemically synthesized and the numbers correspond to the peptide identifiers in the ELISA assay in (B). (B) Reactivities of sera from individual H5N1-infected patients (Viet 1–5) or sera from healthy Vietnamese adults against peptides derived from: PB2 (1); PB1 (2–3); PB1-F2 (4–7); PA (8); NP (9–12); M1 (13–16); M2e (17); M2 (18); NS1 (19–20); and NS2 (21).

Panning of GFPDL (FLU-6) expressing inserts from the internal viral proteins (Figure S2A) with the pooled H5N1 convalescent sera resulted in the isolation of many clones across all proteins (Table S1). The majority of the fragments recognized in the internal proteins were shorter than the HA/NA epitopes (Figure 5A). As expected, most of the selected clones were derived from the major structural proteins M1 and NP (531 and 91 clones, respectively, Table S1). Peptides that were recognized at high frequencies were synthesized and tested with individual sera in ELISA (Figure 5B). Interestingly, binding to all four M1 peptides remained at high mean end-point titers (1∶2,500). Control Vietnamese pooled sera reacted weakly with peptides 10 (NP) and 14–15 (M1). The US control sera reacted strongly with M1 peptides 13–16, reflecting the high degree of conservation in these proteins between seasonal human influenza and H5N1 viruses [7]. As indicated before, student's *t*-test analyses (two-tailed distribution with equal variance and a degree of freedom of 13) revealed that the ELISA reactivities against most of the H5N1 peptides were significantly different (*p*<0.05) between the convalescent sera and the plasma from unexposed US and Vietnamese controls, with the exception of binding to selected peptides derived from PA, NP, and M1 (*p*>0.05 are shown in bold in Table S2, and unpublished data).

The M2 ectodomain (M2e) was postulated to contribute to protective immune responses [18], but evidence for anti-M2e responses in humans is lacking. In our study, a strong reactivity against the M2e peptide (17) was found in H5N1 convalescent sera but not with control sera from either Vietnam or the US (Figure 5B; Table S2).

PB1-F2 is a 90-aa protein encoded by the PB1 gene. It was identified as a potential virulence factor in the 1918 pandemic H1N1 strain, and in the HP H5N1 (HK/97) viruses [19]. However, evidence for expression of this protein during human infection is lacking. Importantly, the pooled H5N1 convalescent sera selected 35 PB1-F2 phage clones (Table S1), and the synthetic PB1-F2 peptides were recognized by each individual convalescent serum, but not by the Vietnamese or US control sera (Figure 5B, peptides 4–7; Table S2). This is the first report of antibodies against PB1-F2 in H5N1-recovered individuals, and strongly suggests that PB1-F2 is expressed during infection with H5N1 viruses.

### HA [(-10)-223] Can Adsorb Virus-Neutralizing Activity in Polyclonal Sera

To evaluate the functional significance of the main HA epitopes identified using GFPDL, we evaluated the ability of the HA peptide [(-10)-223] (that bound strongly to the H5N1-neutralizing MAbs, FLA5.10 and FLD21.140) to block viral neutralization using hyperimmune sheep sera raised against the reassortant A/Vietnam/1203/04 × PR8 virus that had a titer of 1∶640 in a microneutralization assay using the rgA/Vietnam/1203/2004 virus (Table 1). After incubation with HA [(-10)-223], the unbound antibody fraction lost neutralizing activity (titer <1∶40), while the antibodies eluted from the resin-bound HA protein retained neutralizing activity (Table 1, top panel). Incubating the anti-H5N1 sheep serum with GST-His_6_ did not adsorb any neutralizing activity. Additionally, none of the shorter peptides within the HA [(-10)-223], alone or in combination, adsorbed any neutralizing activity. Similar results were obtained with sera from ferrets infected with wild-type A/Vietnam/1203/2004 (H5N1) virus (obtained from Ruben Donis, CDC) (Table 1, lower panel). These results demonstrate for the first time that an HA-1 peptide, expressed in bacteria, can adsorb the majority of anti-H5N1 neutralizing antibodies generated in two animal species, suggesting that although most neutralizing epitopes are conformation dependent, proper folding and presentation of such epitopes is not necessarily dependent on post-translational modifications restricted to eukaryotic cells.

**Table 1:** Adsorption of neutralization activity using HA peptides.

Sera	Peptides Added	Titer
**Sheep anti-A/Vietnam/1203/04 sera**	No peptide	640
	HA [(-10)-223]-FLOW-THROUGH	<40
	HA [(-10)-223]-ELUATE	640
	GST-His-FLOW-THROUGH	640
	GST-His-ELUATE	<40
	HA 35-96+HA 99-121 FLOW-THROUGH	640
	HA 120-149+HA 185-206 FLOW-THROUGH	640
	HA 491-534 FLOW-THROUGH	640
**Ferret anti-A/Vietnam/1203/04-infected sera (2004-53)**	No peptide	640
	HA [(-10)-223] FLOW-THROUGH	<40
	HA 35-96+HA 99-121 FLOW-THROUGH	640
	HA 120-149+HA 185-206 FLOW-THROUGH	640
	HA 491-534 FLOW-THROUGH	640

HA [(-10)-223] can adsorb most of the neutralizing activity in polyclonal sera. Sera from sheep hyperimmunized with bromelain-treated rgA/Vietnam/1203/2004 (FDA), or from ferrets infected with wild-type A/Vietnam/1203/2004 virus (CDC) were adsorbed on different H5 HA peptides and subjected to a microneutralization assay performed with rgA/Vietnam/1203/2004 virus. End-point neutralization titers of various pre- or postadsorbed sera are shown.

## Discussion

GFPDL are a powerful tool to decipher all the primary antigenic sites in influenza viruses following natural exposure or vaccination. Previously, this approach led to the development of HIV-SELECTEST for differential diagnosis of HIV infections in vaccine recipients [9], [20]. In the current study, we used H5N1 GFPDL to identify recognition sites of antibodies in convalescent sera obtained from five Vietnamese individuals with a history of H5N1 infection and two H5-specific neutralizing MAbs derived from two of these survivors. The study identified antibodies targeting the HA, NA, and the M2e among other proteins. Furthermore we provide evidence that the PB1-F2, a putative viral virulence factor, was recognized by antibodies in all five convalescent sera of H5N1-infected individuals (mean end-point titers of ≥1∶500).

Previously, murine MAbs and escape mutants were used to map binding sites on the structures of human influenza HA [15], [16], [21]–[23], and more recently, of H5 HA [24], [25]. In contrast, until recently, very limited information was available on human antibody epitopes in type A viruses, and even less for avian H5N1 viruses [7].

Elucidating the repertoire of influenza-specific human antibodies against all the viral proteins is desired for understanding virus-host interactions and identifying new targets for protection. To that end, we used influenza GFPDL to unravel the antibody specificities of five H5N1-recovered individuals and to map the epitopes of two neutralizing MAbs derived from their memory B cells. Our main findings were: (1) H5N1 convalescent sera contained highly diverse antibody specificities against HA1, HA2, NA, and internal proteins of the virus for at least 6 mo; (2) two human MAbs, which were protective in mice, recognized conformation-dependent nonlinear epitopes in large HA1 fragments that encompass the RBS; (3) the HA1[(-10)-223)] protein showed high avidity binding to the two human MAbs and adsorbed a significant proportion of the neutralizing activity of polyclonal anti-H5N1 sheep and ferret sera; (4) strong antibody reactivity against the NA catalytic site and M2e were identified; (5) convalescent sera bound PB1-F2 peptides providing evidence that this protein is expressed during acute H5N1 infection; (6) sera from 20 Vietnamese adults with no history of H5N1 infection, and from ten US human influenza-confirmed infections, revealed very low reactivity to most of the H5N1 epitopes.

Deciphering the epitopes of the H5-specific human MAbs explained the restricted neutralization pattern of FLA5.10 compared with FLD21.140. MAb FLA5.10 binding requires L129, a critical amino acid within the RBS. A L_129_S change was reported in clade 2 H5N1 viruses from China and Southeast Asia in 2002–2005 [26], and could explain the clade 1-restricted protection provided by FLA5.10. Preliminary data from escape mutants (ALS and KS) support the FLA5.10 epitope identified by phage display libraries (unpublished data). On the other hand, the predicted contact residues for FLD21.140 are highly conserved among clade 1 and clade 2 H5N1 viruses and the high binding affinity of this MAb for the HA [(-10)-223)] fragment may explain its broad cross-protection in vivo. Importantly, for both MAbs the combined GFPDL/RPL approach identified noncontinuous conformation dependent epitopes.

Panning of GFPDL (HA+NA) with sera from individuals who had recovered from H5N1 virus infection revealed broad antibody reactivity against both HA1 and HA2 domains, including the large HA1 fragments bound by the protective human MAbs. Epitope profiling of HA led to identification of six antigenic clusters (I–VI). HA antigenic sites “a–e” were defined primarily using mouse monoclonal antibodies that are encompassed in clusters-I and -II, described in this study.

A recent paper by Throsby et al., describes heterosubtypic neutralizing MAbs that cross react against H5N1 and H1N1 [27]. The epitopes of these monoclonal antibodies were mapped to amino acids 43–58 in HA2. This sequence corresponds to H5-HA-2723-2378 in our study, and was part of several peptide sequences displayed on the affinity selected phage clones (Figure 3B, peptides 10–12; Table S1). While all five H5N1 recovered individuals had titers against these HA2 peptides (reciprocal titers 500–2,500), binding of control plasma (from Vietnam or the US) to these peptides was observed at low frequency, and the titers did not exceed 1∶100 (Figure 3B; Table S2).

Among the H5N1 convalescent plasma, most ELISA reciprocal titers were lower in the 6-mo postinfection sample. However, HA2 peptide (H5-HA-2838-2866) located in the membrane proximal domain was strongly recognized by all convalescent sera (Figure 3B), but did not bind control sera from Vietnam or from US individuals with recent culture-confirmed seasonal influenza infections. This highly conserved HA2 region contains aa changes between H5, H1, and H3 viruses [28] and could be useful for surveillance of H5N1 infections in endemic areas.

This study also identified a strong binding by the H5N1 convalescent sera to a 178-aa fragment containing the NA catalytic site that has not been reported previously. This NA segment was not recognized by US control sera with high HI titers against H1N1. Therefore, we did not find evidence that repeated exposure to human H1N1 influenza viruses elicits high-titer antibodies against the heterologous avian N1 NA, as was previously predicted [29].

Studies in mice have shown that serum anti-M2e antibodies can reduce virus replication and death. Currently, M2 is being evaluated as a component of several “universal vaccines”[30]–[33], and possibly as target for monoclonal antibody therapies [32], [34]. However, the immunogenicity of M2e during natural influenza infections is still debated [35]. Surprisingly, the GFPDL analyses revealed high titer anti-M2e antibodies in post-H5N1 infection sera (but not in control sera). Although the N-terminal (1–9 aa) sequence of M2e is highly conserved across different influenza-A subtypes, there is significant diversity in the C-terminal (10–21 aa) sequence of M2e (http://www.flu.lanl.gov). Also, similar GFPDL analyses using the pooled sera from 20 control H5N1-uninfected Vietnamese females did not select any phage-displaying peptide sequence from the M2 region. This result suggests that strong antibody response is generated against M2e following primary infection with HP H5N1 strain. Thus, the contribution of M2e antibodies to viral clearance after H5N1 infection should be further evaluated.

PB1-F2, a recently discovered proapoptotic influenza-A viral protein, contributes to viral pathogenesis in mice [36]. However, this protein is not incorporated into viral particles, and evidence for its expression during influenza infection of animals or humans is lacking. In our study, 35 PB1-F2–expressing GFPDL clones were isolated, and sera from all five H5N1 infection survivors, but not control sera, reacted with synthetic PB1-F2 peptides in ELISA. To our knowledge, this is the first report indicating that PB1-F2 is expressed during H5N1 virus infection in humans. It is not clear how this short-lived protein activates B cells. However, high viral replication and cell lysis during H5N1 infection could present the newly expressed viral proteins to the immune system.

The use of GFPDL to decipher the complete B cell repertoires in AIV-exposed individuals is limited by the fact that all protein segments are expressed in a bacterial system. Therefore, epitopes that are strictly dependent on post-translational modifications or on the multimeric forms of influenza proteins in the native structure might have been missed in our analyses. In spite of these limitations, the use of GFPDL led to new insights into the repertoire of anti-influenza antibodies following H5N1 virus infection and of epitopes that may contribute to resolution of avian influenza infections and could be incorporated into future vaccines. Finally, conserved epitopes recognized by sera from convalescent individuals may be useful for monitoring outbreaks of avian influenza.

## Supporting Information

Figure S1 The complete H5N1 A/Vietnam/1203/04 proteome sequence was constructed by linking the 11 proteins (protein names are shown within the proteome sequence) coded by the eight gene segments derived from wild-type A/Vietnam/1203/2004 viral RNA grown in embryonated chicken eggs. The predicted glycosylation sites (NXT/NXS) in HA are underlined.
(0.04 MB DOC)

Figure S2 Construction of H5N1 A/Vietnam phage display libraries: size and insert distribution. (A) Four phage display libraries for the H5N1 strain A/Vietnam/1203/2004 were constructed: fSK9-3 H5Viet-HA-NA (50–200-bp inserts); fSK9-3 H5Viet-HA-NA (200–1,000-bp inserts); fSK9-3 H5Viet-FLU-6 (50–200-bp inserts) (PB2, PB1, PA, NP, M, and NS genes); and fSK9-3 H5Viet- FLU-6 PB2-NS (200–1,000-bp inserts). Each library size was calculated by plating serial dilutions of *E. coli* transformants following electroporation. (B) Sequence diversity of inserts in complete H5N1 gene-fragment phage display library. 190 colonies from each library were subjected to PCR-based sequencing. Representative alignment of A/Vietnam/1203/2004 genome sequence with inserts in combined (fSK9-3 H5Viet-HA-NA [50–200 bp] and fSK9-3 H5Viet- FLU-6 PB2-NS [(50–200 bp]) gene-fragment phage display library is shown. Arrow indicates the orientation of inserts. Similar distribution of inserts was found for the fSK9-3 H5Viet-HA-NA (200–1,000 bp) and fSK9-3 Viet- FLU-6 PB2-NS (200–1,000 bp). All open reading frames of the influenza-coded proteins were represented in the GFPDL with good representation of smaller and larger inserts in both the transfected bacteria and the phage libraries.
(0.75 MB EPS)

Figure S3 Binding of MAb FLA5.10 to peptide 5.10-101 (identified using RPL panning) compared with mutated peptide 5.10-101-L1A in ELISA. Both biotinylated peptides were captured on streptavidin-coated plates and reacted with serial dilutions of FLA5.10. Leucine to alanine substitution resulted in >98% loss of FLA5.10 binding.
(0.37 MB EPS)

Figure S4 Adsorption of anti-HA antibodies in convalescent sera from survivors of H5N1 infection using the H5N1 GFPDL. Pooled sera from five H5N1 survivors were adsorbed with H5 (HA+NA) GFPDL. Binding to recombinant HA protein (A/Vietnam/1203/2004, Protein Sciences Corporation) is shown before (circles) and after (triangles) GFPDL-adsorption.
(0.38 MB EPS)

Table S1 Frequency of selected phage clones using H5N1 GFPDL after panning with sera from five H5N1-Vietnam infection survivors. Three rounds of affinity selection were performed on pooled sera using each of the four GFPDL under both conditions (antibody coated beads and in-solution). 48 clones were sequenced in each panning round, resulting in sequencing of 2,304 total clones. The peptide sequences displayed on the phage surface and the corresponding frequencies for these phage displayed sequences are shown. Each peptide name indicates the H5N1 protein name and the amino acid numbers corresponding to the complete proteome sequence shown in Figure S1. Sequences in bold letters represent peptides that were used for synthesis and follow up binding assays.
(0.14 MB DOC)

Table S2 ELISA reactivity of sera from ten individuals with culture-confirmed seasonal influenza infections during the 2006–2007 seasons. End-point antibody titers (based on 5-fold dilutions starting at 1∶100) are reported for US 1–10 against the identical H5N1-Viet peptides used in Figures 4B and 6B). Student *t*-test was performed for each peptide reactivity using the end-point titers for the control sera (e.g., if end-point titer was <100, the value used for statistical analysis was 20), compared with the end-point titers of the five H5N1-convelescent samples (e.g., if end-point titer was >12,500, the value used for statistical analysis was 12,500). *p*-Values appear in the right column.
(0.15 MB DOC)

## Abbreviations

aa: amino acid
AIV: avian influenza viruses
CDC: Centers for Disease Control
GFPDL: whole-genome–fragment phage display libraries
HA: haemagglutinin
HP: highly pathogenic
M2e: M2 ectodomain
MAb: human monoclonal antibodies
NA: neuraminidase
RBS: receptor binding site
RPL: random peptide phage display library
RT: room temperature
