# Supplementary material for: Antigenic Fingerprinting of H5N1 Avian Influenza Using Convalescent Sera and Monoclonal Antibodies Reveals Potential Vaccine and Diagnostic Targets
Source: PLoS Med. 2009 Apr 21;6(4):e1000049. doi: 10.1371/journal.pmed.1000049 (PMC2661249; doi:10.1371/journal.pmed.1000049)
Supplement: Figure S1 — The complete H5N1 A/Vietnam/1203/04 proteome sequence was constructed by linking the 11 proteins (protein names are shown within the proteome sequence) coded by the eight gene segments derived from wild-type A/Vietnam/1203/2004 viral RNA grown in embryonated chicken eggs. The predicted glycosylation sites (NXT/NXS) in HA are underlined. (0.04 MB DOC) [file pmed.1000049.s001.doc]

**COMPLETE PROTEOME SEQUENCE OF H5N1- A/VIETNAM/1203/2004**

Sequence Range: 1 to 4552

10 20 30 40 50 60 70 80 90 100

MERIKELRDLMSQSRTREILTKTTVDHMAIIKKYTSGRQEKNPALRMKWMMAMKYPITADKRIIEMIPERNEQGQTLWSKTNDAGSDRVMVSPLAVTWWN

______________________________________POLYMERASE PROTEIN-PB2________________________________________>

110 120 130 140 150 160 170 180 190 200

RNGPATSAVHYPKVYKTYFEKVERLKHGTFGPVHFRNQVKIRRRVDINPGHADLSAKEAQDVIMEVVFPNEVGARILTSESQLTITKEKKEELQDCKIAP

______________________________________POLYMERASE PROTEIN-PB2________________________________________>

210 220 230 240 250 260 270 280 290 300

LMVAYMLERELVRKTRFLPVAGGTSSVYIEVLHLTQGTCWEQMYTPGGEVRNDDVDQSLIIAARNIVRRATVSADPLASLLEMCHSTQIGGIRMVDILRQ

______________________________________POLYMERASE PROTEIN-PB2________________________________________>

310 320 330 340 350 360 370 380 390 400

NPTEEQAVDICKAAMGLRISSSFSFGGFTFKRTSGSSVKKEEEVLTGNLQTLKIRVHEGYEEFTMVGRRATAILRKATRRLIQLIVSGRDQQSIAEAIIV

______________________________________POLYMERASE PROTEIN-PB2________________________________________>

410 420 430 440 450 460 470 480 490 500

AMVFSQEDCMIKAVRGDLNFVNRANQRLNPMHQLLRHFQKDAKVLFQNWGIEPIDNVMGMIGILPDMTPSTEMSLRGVRVSKMGVDEYSSTERVVVSIDR

______________________________________POLYMERASE PROTEIN-PB2________________________________________>

510 520 530 540 550 560 570 580 590 600

FLRVRDQRGNVLLSPEEVSETQGTEKLTITYSSSMMWEINGPESVLVNTYQWIIRNWETVKIQWSQDPTMLYNKMEFEPFQSLVPKAARGQYSGFVRTLF

______________________________________POLYMERASE PROTEIN-PB2________________________________________>

610 620 630 640 650 660 670 680 690 700

QQMRDVLGTFDTVQIIKLLPFAAAPPKQSRMQFSSLTVNVRGSGMRILVRGNSPVFNYNKATKRLTVLGKDAGALTEDPDEGTAGVESAVLRGFLILGKE

______________________________________POLYMERASE PROTEIN-PB2________________________________________>

710 720 730 740 750 760 770 780 790 800

DKRYGPALSINELSNLAKGEKANVLIGQGDVVLVMKRKRDSSILTDSQTATKRIRMAIN---MDVNPTLLFLKVPVQNAISTTFPYTGDPPYSHGTGTGY

__________________POLYMERASE PROTEIN-PB2____________________>

_______POLYMERASE PROTEIN-PB1_________>

810 820 830 840 850 860 870 880 890 900

TMDTVNRTHQYSEKGKWTTNTETGAPQLNPIDGPLPEDNEPSGYAQTDCVLEAMAFLEESHPGIFENSCLETMEIVQQTRVDKLTQGRQTYDWTLNRNQP

______________________________________POLYMERASE PROTEIN-PB1________________________________________>

910 920 930 940 950 960 970 980 990 1000

AATALANTIEIFRSNGLTANESGRLIDFLKDVMESMDKEEMEITTHFQRKRRVRDNMTKKMVTQRTIGXKKQRLNKKSYLIRALTLNTMTKDAERGKLKR

______________________________________POLYMERASE PROTEIN-PB1________________________________________>

1010 1020 1030 1040 1050 1060 1070 1080 1090 1100

RAIATPGMQIRGFVYFVETLARSICEKLEQSGLPVGGNEKKAKLANVVRKMMTNSQDTELSFTITGDNTKWNENQNPRMFLAMITYITRNQPEWFRNVLS

______________________________________POLYMERASE PROTEIN-PB1________________________________________>

1110 1120 1130 1140 1150 1160 1170 1180 1190 1200

IAPIMFSNKMARLGKGYMFESKSMKLRTQIPAEMLANIDLKYFNELTKKKIEKIRPLLIDGTASLSPGMMMGMFNMLSTVLGVSILNLGQKRYTKTTXWW

______________________________________POLYMERASE PROTEIN-PB1________________________________________>

1210 1220 1230 1240 1250 1260 1270 1280 1290 1300

DGLQSSDDFALIVNAPNHEGIQAGVDRFYRTCKLVGINMSKKKSYINRTGTFEFTSFFYRYGFVANFSMELPSFGVSGINESADMSIGVTVIKNNMINND

______________________________________POLYMERASE PROTEIN-PB1________________________________________>

1310 1320 1330 1340 1350 1360 1370 1380 1390 1400

LGPATAQMALQLFIKDYRYTYRCHRGDTQIQTRRSFELKKLWEQTRSKAGLLVSDGGPNLYNIRNLHIPEVCLKWELMDEDYQGRLCNPLNPFVSHKEIE

______________________________________POLYMERASE PROTEIN-PB1________________________________________>

1410 1420 1430 1440 1450 1460 1470 1480 1490 1500

SVNNAVVMPAHGPAKSMEYDAVATTHSWIPKRNRSILNTSQRGILEDEQMYQKCCNLFEKFFPSSSYRRPVGISSMVEAMVSRARIDARIDFESGRIKKE

______________________________________POLYMERASE PROTEIN-PB1________________________________________>

1510 1520 1530 1540 1550 1560 1570 1580 1590 1600

EFAEIMKICSTIEELRRQK---MEQGQDTPWTQSTEHTNIQKRGSGQQTQRLEHPNSTRLMDHYLRIMSPVGTHKQIVYWKQWLSLKNPTQGSLKTRVLK

___POLYMERASE P____>

___________________________________PB1-F2_____________________________________>

1610 1620 1630 1640 1650 1660 1670 1680 1690 1700

RWKLFNKQEWIN---MEDFVRQCFNPMIVELAEKAMKEYGEDPKIETNKFAAICTHLEVCFMYSDFHFIDERSESIIVESGDPNALLKHRFEIIEGRDRT

____________>

_______________________________POLYMERASE PROTEIN-PA_________________________________>

1710 1720 1730 1740 1750 1760 1770 1780 1790 1800

MAWTVVNSICNTTGVEKPKFLPDLYDYKENRFIEIGVTRREVHTYYLEKANKIKSEETHIHIFSFTGEEMATKADYTLDEESRARIKTRLFTIRQEMASR

_______________________________________POLYMERASE PROTEIN-PA________________________________________>

1810 1820 1830 1840 1850 1860 1870 1880 1890 1900

GLWDSFRQSERGEETIEEKFEITGTMRRLADQSLPPNFSSLENFRAYVDGFEPNGCIEGKLSQMSKEVNARIEPFLKTTPRPLRLPDGPPCSQRSKFLLM

_______________________________________POLYMERASE PROTEIN-PA________________________________________>

1910 1920 1930 1940 1950 1960 1970 1980 1990 2000

DALKLSIEDPSHEGEGIPLYDAIKCMKTFFGWKEPNIVKPHEKGINPNYLLAWKQVLAELQDIENEEKIPKTKNMKKTSQLKWALGENMAPEKVDFEDCK

_______________________________________POLYMERASE PROTEIN-PA________________________________________>

2010 2020 2030 2040 2050 2060 2070 2080 2090 2100

DVSDLRQYDSDEPESRSLASWIQSEFNKACELTDSIWIELDEIGEDVAPIEHIASMRRNYFTAEVSHCRATEYIMKGVYINTALLNASCAAMDDFQLIPM

_______________________________________POLYMERASE PROTEIN-PA________________________________________>

2110 2120 2130 2140 2150 2160 2170 2180 2190 2200

ISKCRTKEGRRKTNLYGFIIKGRSHLRNDTDVVNFVSMEFSLTDPRLEPHKWEKYCVLEIGDMLLRTAVGQVSRPMFLYVRTNGTSKIKMKWGMEMRRCL

_______________________________________POLYMERASE PROTEIN-PA________________________________________>

2210 2220 2230 2240 2250 2260 2270 2280 2290 2300

LQSLQQIESMIEAESSVKEKDMTKEFFENKSETWPIGESPKGVEEGSIGKVCRTLLAKSVFNSLYASPQLEGFSAESRKLLLIAQALRDNLEPGTFDLGG

_______________________________________POLYMERASE PROTEIN-PA________________________________________>

2310 2320 2330 2340 2350 2360 2370 2380 2390 2400

LYEAIEECLINDPWVLLNASWFNSFLAHALK---MEKIVLLFAIVSLVKSDQICIGYHANNSTEQVDTIMEKNVTVTHAQDILEKKHNGKLCDLDGVKPL

____POLYMERASE PROTEIN-PA______>

________________________HAEMAGGLUTININ-HA_________________________>

2410 2420 2430 2440 2450 2460 2470 2480 2490 2500

ILRDCSVAGWLLGNPMCDEFINVPEWSYIVEKANPVNDLCYPGDFNDYEELKHLLSRINHFEKIQIIPKSSWSSHEASLGVSSACPYQGKSSFFRNVVWL

_________________________________________HAEMAGGLUTININ-HA__________________________________________>

2510 2520 2530 2540 2550 2560 2570 2580 2590 2600

IKKNSTYPTIKRSYNNTNQEDLLVLWGIHHPNDAAEQTKLYQNPTTYISVGTSTLNQRLVPRIATRSKVNGQSGRMEFFWTILKPNDAINFESNGNFIAP

_________________________________________HAEMAGGLUTININ-HA__________________________________________>

2610 2620 2630 2640 2650 2660 2670 2680 2690 2700

EYAYKIVKKGDSTIMKSELEYGNCNTKCQTPMGAINSSMPFHNIHPLTIGECPKYVKSNRLVLATGLRNSPQRERRRKKRGLFGAIAGFIEGGWQGMVDG

_________________________________________HAEMAGGLUTININ-HA__________________________________________>

2710 2720 2730 2740 2750 2760 2770 2780 2790 2800

WYGYHHSNEQGSGYAADKESTQKAIDGVTNKVNSIIDKMNTQFEAVGREFNNLERRIENLNKKMEDGFLDVWTYNAELLVLMENERTLDFHDSNVKNLYD

_________________________________________HAEMAGGLUTININ-HA__________________________________________>

2810 2820 2830 2840 2850 2860 2870 2880 2890 2900

KVRLQLRDNAKELGNGCFEFYHKCDNECMESVRNGTYDYPQYSEEARLKREEISGVKLESIGIYQILSIYSTVASSLALAIMVAGLSLWMCSNGSLQCRI

_________________________________________HAEMAGGLUTININ-HA__________________________________________>

2910 2920 2930 2940 2950 2960 2970 2980 2990 3000

CI---MASQGTKRSYEQMETGGERQNATEIRASVGRMVSGIGRFYIQMCTELKLSDYEGRLIQNSITIERMVLSAFDERRNRYLEEHPSAGKDPKKTGGP

__>

_______________________________________NUCLEOPROTEIN-NP________________________________________>

3010 3020 3030 3040 3050 3060 3070 3080 3090 3100

IYRRRDGKWVRELILYDKEEIRRIWRQANNGEDATAGLTHLMIWHSNLNDATYQRTRALVRTGMDPRMCSLMQGSTLPRRSGAAGAAVKGVGTMVMELIR

_________________________________________NUCLEOPROTEIN-NP___________________________________________>

3110 3120 3130 3140 3150 3160 3170 3180 3190 3200

MIKRGINDRNFWRGENGRRTRIAYERMCNILKGKFQTAAQRAMMDQVRESRNPGNAEIEDLIFLARSALILRGSVAHKSCLPACVYGLAVASGYDFEREG

_________________________________________NUCLEOPROTEIN-NP___________________________________________>

3210 3220 3230 3240 3250 3260 3270 3280 3290 3300

YSLVGIDPFRLLQNSQVFSLIRPNENPAHKSQLVWMACHSAAFEDLRVSSFIRGTRVVPRGQLSTRGVQIASNENMEAMDSNTLELRSRYWAIRTRSGGN

_________________________________________NUCLEOPROTEIN-NP___________________________________________>

3310 3320 3330 3340 3350 3360 3370 3380 3390 3400

TNQQRASAGQISVQPTFSVQRNLPFERATIMAAFTGNTEGRTSDMRTEIIRMMESARPEDVSFQGRGVFELSDEKATNPIVPSFDMNNEGSYFFGDNAEE

_________________________________________NUCLEOPROTEIN-NP___________________________________________>

3410 3420 3430 3440 3450 3460 3470 3480 3490 3500

YDN---MNPNQKIITIGSICMVTGIVSLMLQIGNMISIWVSHSIHTGNQHQSEPISNTNFLTEKAVASVKLAGNSSLCPINGWAVYSKDNSIRIGSKGDV

___>

______________________________________NEURAMINADASE-NA________________________________________>

3510 3520 3530 3540 3550 3560 3570 3580 3590 3600

FVIREPFISCSHLECRTFFLTQGALLNDKHSNGTVKDRSPHRTLMSCPVGEAPSPYNSRFESVAWSASACHDGTSWLTIGISGPDNGAVAVLKYNGIITD

_________________________________________NEURAMINADASE-NA___________________________________________>

3610 3620 3630 3640 3650 3660 3670 3680 3690 3700

TIKSWRNNILRTQESECACVNGSCFTVMTDGPSNGQASHKIFKMEKGKVVKSVELDAPNYHYEECSCYPNAGEITCVCRDNWHGSNRPWVSFNQNLEYQI

_________________________________________NEURAMINADASE-NA___________________________________________>

3710 3720 3730 3740 3750 3760 3770 3780 3790 3800

GYICSGVFGDNPRPNDGTGSCGPVSSNGAYGVKGFSFKYGNGVWIGRTKSTNSRSGFEMIWDPNGWTETDSSFSVKQDIVAITDWSGYSGSFVQHPELTG

_________________________________________NEURAMINADASE-NA___________________________________________>

3810 3820 3830 3840 3850 3860 3870 3880 3890 3900

LDCIRPCFWVELIRGRPKESTIWTSGSSISFCGVNSDTVGWSWPDGAELPFTIDK---MSLLTEVETYVLSIIPSGPLKAEIAQKLEDVFAGKNTDLEAL

___________________NEURAMINADASE-NA____________________>

____________MATRIX PROTEIN-M1_____________>

3910 3920 3930 3940 3950 3960 3970 3980 3990 4000

MEWLKTRPILSPLTKGILGFVFTLTVPSERGLQRRRFVQNALNGNGDPNNMDRAVKLYKKLKREITFHGAKEVALSYSTGALASCMGLIYNRMGTVTTEV

_________________________________________MATRIX PROTEIN-M1__________________________________________>

4010 4020 4030 4040 4050 4060 4070 4080 4090 4100

AFGLVCATCEQIADSQHRSHRQMATITNPLIRHENRMVLASTTAKAMEQMAGSSEQAAEAMEIANQARQMVQAMRTIGTHPNSSAGLRDNLLENLQAYQK

_________________________________________MATRIX PROTEIN-M1__________________________________________>

4110 4120 4130 4140 4150 4160 4170 4180 4190 4200

RMGVQMQRFK---MSLLTEVETPTRNEWECRCSDSSDPIVVAANIIGILHLILWILDRLFFKCIYRRLKYGLKRGPATAGVPESMREEYRQEQQSAVDVD

__________>

__________________________________________M2___________________________________________>

4210 4220 4230 4240 4250 4260 4270 4280 4290 4300

DGHFVNIELE---MDSNTVSSFQVDCFLWHVRKRFADQELGDAPFLDRLRRDQKSLRGRGNTLGLDIETATRAGKQIVERILEGESDKALKMPASRYLTD

__________>

______________________________NON-STRUCTURAL PROTEIN-NS1_______________________________>

4310 4320 4330 4340 4350 4360 4370 4380 4390 4400

MTLEEMSRDWFMLMPKQKVAGSLCIKMDQAIMDKTIILKANFSVIFDRLETLILLRAFTEEGAIVGEISPLPSLPGHTGEDVKNAIGVLIGGLEWNDNTV

____________________________________NON-STRUCTURAL PROTEIN-NS1______________________________________>

4410 4420 4430 4440 4450 4460 4470 4480 4490 4500

RVTETIQRFAWRNSDEDGRLPLPPNQKR---MDSNTVSSFQDILVRMSKMQLASSSEDLNGMITQFESLKLYRDSLGETVMRMGDFHSLQIRNGKWREQL

___NON-STRUCTURAL PROTEI____>

_____________________NON-STRUCTURAL PROTEIN-NS2______________________>

4510 4520 4530 4540 4550

SQKFEEIRWLIEEVRHRLKITENSFEQITFMQALQLLLEVEQEIRAFSFQLI

____________NON-STRUCTURAL PROTEIN-NS2______________>
