# Supplementary material for: Antigenic Fingerprinting of H5N1 Avian Influenza Using Convalescent Sera and Monoclonal Antibodies Reveals Potential Vaccine and Diagnostic Targets
Source: PLoS Med. 2009 Apr 21;6(4):e1000049. doi: 10.1371/journal.pmed.1000049 (PMC2661249; doi:10.1371/journal.pmed.1000049)
Supplement: Table S1 — Frequency of selected phage clones using H5N1 GFPDL after panning with sera from five H5N1-Vietnam infection survivors. Three rounds of affinity selection were performed on pooled sera using each of the four GFPDL under both conditions (antibody coated beads and in-solution). 48 clones were sequenced in each panning round, resulting in sequencing of 2,304 total clones. The peptide sequences displayed on the phage surface and the corresponding frequencies for these phage displayed sequences are shown. Each peptide name indicates the H5N1 protein name and the amino acid numbers corresponding to the complete proteome sequence shown in Figure S1. Sequences in bold letters represent peptides that were used for synthesis and follow up binding assays. (0.14 MB DOC) [file pmed.1000049.s005.doc]

**SUPPLEMENTARTY TABLE-1**

FREQUENCY OF SELECTED PHAGE CLONES WITH H5N1 EXPOSED SURVIVORS USING H5N1 GENE-FRAGMENT PHAGE DISPLAY LIBRARIES

| **Influenza Peptide Name** | **PEPTIDE SEQUENCE** | **FREQUENCY** |
| --- | --- | --- |
| **H5-PB2-344-375** | **EVLTGNLQTLKIRVHEGYEEFTMVGRRATAILR** | **9** |
| H5-PB2-447-516 | QNWGIEPIDNVMGMIGILPDMTPSTEMSLRGVRVSKMGVDEYSSTERVVVSIDRFLRVRDQRGNVLLSPE | 2 |
| H5-PB1-1290-1451 | TVIKNNMINNDLGPATAQMALQLFIKDYRYTYRCHRGDTQIQTRRSFELKKLWEQTRSKAGLLVSDGGPNLYNIRNLHIPEVCLKWELMDEDYQGRLCNPLNPFVSHKEIEVNNAVVMPAHGPAKSMEYDAVATTHSWIPKRNRSILNTSQRGILEDEQMY | 2 |
| **H5-PB1-1348-1361** | **KAGLLVSDGGPNLY** | **7** |
| **H5-PB1-1420-1437** | **DAVATTHSWIPKRNRSIL** | **6** |
| **H5-PB1-F2-1524-1598** | **EQGQDTPWTQSTEHTNIQKRGSGQQTQRLEHPNSTRLMDHYLRIMSPVGTHKQIVYWKQWLSLKNPTQGSLKTR** | **13** |
| **H5-PB1-F2-1525-1572** | **QGQDTPWTQSTEHTNIQKRGSGQQTQRLEHPNSTRLMDHYLRIMSPVG** | **5** |
| H5-PB1-F2-1548-1608 | QTQRLEHPNSTRLMDHYLRIMSPVGTHKQIVYWKQWLSLKNPTQGSLKTRVLKRWKLFNKQ | 4 |
| **H5-PB1-F2-1560-1592** | **LMDHYLRIMSPVGTHKQIVYWKQWLSLKNPTQG** | **6** |
| **H5-PB1-F2-1570-1605** | **PVGTHKQIVYWKQWLSLKNPTQGSLKTRVLKRWKLF** | **7** |
| H5-PA-1852-1966 | EPNGCIEGKLSQMSKEVNARIEPFLKTTPRPLRLPDGPPCSQRSKFLLMDALKLSIEDPSHEGEGIPLYDAIKCMKTFFGWKEPNIVKPHEKGINPNYLLAWKQVLAELQDIENE | 3 |
| H5-PA-1904-1927 | KLSIEDPSHEGEGIPLYDAIKCMK | 1 |
| H5-PA-1945-1973 | INPNYLLAWKQVLAELQDIENEEKIPKTK | 1 |
| **H5-PA-2202-2251** | **QSLQQIESMIEAESSVKEKDMTKEFFENKSETWPIGESPKGVEEGSIGKV** | **8** |
| H5-HA-2349-2389 | KSDQICIGYHANNSTEQVDTIMEKNVTVTHAQDILEKKHNG | 1 |
| **H5-HA-2365-2427** | **VDTIMEKNVTVTHAQDILEKKHNGKLCDLDGVKPLILRDCSVAGWLLGNPMCDEFINVPEWS** | **9** |
| **H5-HA-2376-2659** | **VTHAQDILEKKHNGKLCDLDGVKPLILRDCSVAGWLLGNPMCDEFINVPEWSYIVEKANPVNDLCYPGDFNDYEELKHLLSRINHFEKIQIIPKSSWSSHEASLGVSSACPYQGKSSFFRNVVWLIKKNSTYPTIKRSYNNTNQEDLLVLWGIHHPNDAAEQTKLYQNPTTYISVGTSTLNQRLVPRIATRSKVNGQSGRMEFFWTILKPNDAINFESNGNFIAPEYAYKIVKKGDSTIMKSELEYGNCNTKCQTPMGAINSSMPFHNIHPLTIGECPKYVKSN** | **17** |
| **H5-HA-2339-2581** | **VLLFAIVSLVKSDQICIGYHANNSTEQVDTIMEKNVTVTHAQDILEKKHNGKLCDLDGVKPLILRDCSVAGWLLGNPMCDEFINVPEWSYIVEKANPVNDLCYPGDFNDYEELKHLLSRINHFEKIQIIPKSSWSSHEASLGVSSACPYQGKSSFFRNVVWLIKKNSTYPTIKRSYNNTNQEDLLVLWGIHHPNDAAEQTKLYQNPTTYISVGTSTLNQRLVPRIATRSKVNGQSGRMEFFWT** | **13** |
| **H5-HA-2431-2453** | **EKANPVNDLCYPGDFNDYEELKH** | **36** |
| H5-HA-2436-2543 | NDYEELKHLLSRINHFEKIQIIPKSSWSSHEASLGVSSACPYQGKSSFFRNVVWLIKKNSTYPTIKRSYNNTNQEDLLVLWGIHHPNDAAEQTKLYQN | 3 |
| **H5-HA-2452-2481** | **KHLLSRINHFEKIQIIPKSSWSSHEASLGV** | **26** |
| H5-HA-2484-2514 | ACPYQGKSSFFRNVVWLIKKNSTYPTIKRSY | 1 |
| **H5-HA-2517-2538** | **TNQEDLLVLWGIHHPNDAAEQT** | **16** |
| H5-HA-2520-2566 | EDLLVLWGIHHPNDAAEQTKLYQNPTTYISVGTSTLNQRLVPRIATR | 9 |
| H5-HA-2568-2649 | KVNGQSGRMEFFWTILKPNDAINFESNGNFIAPEYAYKIVKKGDSTIMKSELEYGNCNTKCQTPMGAINSSMPFHNIHPLTI | 4 |
| H5-HA-2603-2637 | AYKIVKKGDSTIMKSELEYGNCNTKCQTPMGAINS | 3 |
| H5-HA-2614-2639 | IMKSELEYGNCNTKCQTPMGAINSSM | 1 |
| **H5-HA-2627-2669** | **KCQTPMGAINSSMPFHNIHPLTIGECPKYVKSNRLVLATGLRN** | **17** |
| H5-HA-2632-2651 | MGAINSSMPFHNIHPLTIGE | 2 |
| **H5-HA-2642-2685** | **HNIHPLTIGECPKYVKSNRLVLATGLRNSPQRERRRKKRGLFGA** | **9** |
| H5-HA-2648-2670 | TIGECPKYVKSNRLVLATGLRNS | 2 |
| **H5-HA-2682-2703** | **LFGAIAGFIEGGWQGMVDGWYG** | **13** |
| H5-HA-2686-2748 | IAGFIEGGWQGMVDGWYGYHHSNEQGSGYAADKESTQKAIDGVTNKVNSIIDKMNTQFEAVGR | 4 |
| **H5-HA-2695-2756** | **QGMVDGWYGYHHSNEQGSGYAADKESTQKAIDGVTNKVNSIIDKMNTQFEAVGREFNNLERR** | **34** |
| **H5-HA-2703-2731** | **GYHHSNEQGSGYAADKESTQKAIDGVTNK** | **26** |
| H5-HA-2703-2753 | GYHHSNEQGSGYAADKESTQKAIDGVTNKVNSIIDKMNTQFEAVGREFNNL | 3 |
| H5-HA-2706-2808 | HSNEQGSGYAADKESTQKAIDGVTNKVNSIIDKMNTQFEAVGREFNNLERRIENLNKKMEDGFLDVWTYNAELLVLMENERTLDFHDSNVKNLYDKVRLQLRD | 36 |
| H5-HA-2706-2843 | HSNEQGSGYAADKESTQKAIDGVTNKVNSIIDKMNTQFEAVGREFNNLERRIENLNKKMEDGFLDVWTYNAELLVLMENERTLDFHDSNVKNLYDKVRLQLRDNAKELGNGCFEFYHKCDNECMESVRNGTYDYPQYS | 19 |
| H5-HA-2707-2753 | SNEQGSGYAADKESTQKAIDGVTNKVNSIIDKMNTQFEAVGREFNNL | 17 |
| H5-HA-2707-2786 | SNEQGSGYAADKESTQKAIDGVTNKVNSIIDKMNTQFEAVGREFNNLERRIENLNKKMEDGFLDVWTYNAELLVLMENER | 4 |
| H5-HA-2709-2727 | EQGSGYAADKESTQKAIDG | 1 |
| H5-HA-2711-2730 | GSGYAADKESTQKAIDGVTN | 6 |
| H5-HA-2716-2791 | ADKESTQKAIDGVTNKVNSIIDKMNTQFEAVGREFNNLERRIENLNKKMEDGFLDVWTYNAELLVLMENERTLDFH | 3 |
| H5-HA-2722-2749 | QKAIDGVTNKVNSIIDKMNTQFEAVGRE | 8 |
| H5-HA-2724-2756 | AIDGVTNKVNSIIDKMNTQFEAVGREFNNLERR | 12 |
| **H5-HA-2722-2762** | **QKAIDGVTNKVNSIIDKMNTQFEAVGREFNNLERRIENLNK** | **9** |
| H5-HA-2729-2750 | TNKVNSIIDKMNTQFEAVGREF | 5 |
| **H5-HA-2759-2814** | **NLNKKMEDGFLDVWTYNAELLVLMENERTLDFHDSNVKNLYDKVRLQLRDNAKELG** | **54** |
| H5-HA-2805-2860 | QLRDNAKELGNGCFEFYHKCDNECMESVRNGTYDYPQYSEEARLKREEISGVKLES | 2 |
| H5-HA-2805-2828 | QLRDNAKELGNGCFEFYHKCDNEC | 1 |
| H5-HA-2823-2866 | KCDNECMESVRNGTYDYPQYSEEARLKREEISGVKLESIGIYQI | 172 |
| H5-HA-2829-2864 | MESVRNGTYDYPQYSEEARLKREEISGVKLESIGIY | 32 |
| **H5-HA-2838-2866** | **DYPQYSEEARLKREEISGVKLESIGIYQI** | **154** |
| **H5-NP-2906-2929** | **MASQGTKRSYEQMETGGERQNATE** | **8** |
| H5-NP-2915-3011 | YEQMETGGERQNATEIRASVGRMVSGIGRFYIQMCTELKLSDYEGRLIQNSITIERMVLSAFDERRNRYLEEHPSAGKDPKKTGGPIYRRRDGKWVR | 13 |
| H5-NP-2915-3068 | YEQMETGGERQNATEIRASVGRMVSGIGRFYIQMCTELKLSDYEGRLIQNSITIERMVLSAFDERRNRYLEEHPSAGKDPKKTGGPIYRRRDGKWVRELILYDKEEIRRIWRQANNGEDATAGLTHLMIWHSNLNDATYQRTRALVRTGMDPRM | 3 |
| H5-NP-2939-3055 | SGIGRFYIQMCTELKLSDYEGRLIQNSITIERMVLSAFDERRNRYLEEHPSAGKDPKKTGGPIYRRRDGKWVRELILYDKEEIRRIWRQANNGEDATAGLTHLMIWHSNLNDATYQR | 6 |
| **H5-NP-2955-3011** | **SDYEGRLIQNSITIERMVLSAFDERRNRYLEEHPSAGKDPKKTGGPIYRRRDGKWVR** | **26** |
| H5-NP-2964-3022 | NSITIERMVLSAFDERRNRYLEEHPSAGKDPKKTGGPIYRRRDGKWVRELILYDKEEIR | 11 |
| H5-NP-3197-3229 | EREGYSLVGIDPFRLLQNSQVFSLIRPNENPAH | 2 |
| H5-NP-3214-3261 | NSQVFSLIRPNENPAHKSQLVWMACHSAAFEDLRVSSFIRGTRVVPRG | 3 |
| **H5-NP-3263-3305** | **LSTRGVQIASNENMEAMDSNTLELRSRYWAIRTRSGGNTNQQR** | **8** |
| H5-NP-3329-3399 | TIMAAFTGNTEGRTSDMRTEIIRMMESARPEDVSFQGRGVFELSDEKATNPIVPSFDMNNEGSYFFGDNAE | 2 |
| **H5-NP-3347-3385** | **TEIIRMMESARPEDVSFQGRGVFELSDEKATNPIVPSFD** | **9** |
| **H5-NA-3431-3481** | **QIGNMISIWVSHSIHTGNQHQSEPISNTNFLTEKAVASVKLAGNSSLCPIN** | **7** |
| H5-NA-3453-3480 | EPISNTNFLTEKAVASVKLAGNSSLCPI | 3 |
| H5-NA-3469-3504 | VKLAGNSSLCPINGWAVYSKDNSIRIGSKGDVFVIR | 2 |
| **H5-NA-3489-3530** | **DNSIRIGSKGDVFVIREPFISCSHLECRTFFLTQGALLNDKH** | **11** |
| H5-NA-3522-3541 | QGALLNDKHSNGTVKDRSPH | 1 |
| **H5-NA-3541-3576** | **HRTLMSCPVGEAPSPYNSRFESVAWSASACHDGTSW** | **9** |
| H5-NA-3548-3613 | PVGEAPSPYNSRFESVAWSASACHDGTSWLTIGISGPDNGAVAVLKYNGIITDTIKSWRNNILRTQ | 1 |
| H5-NA-3578-3606 | TIGISGPDNGAVAVLKYNGIITDTIKSWR | 2 |
| H5-NA-3618-3650 | ACVNGSCFTVMTDGPSNGQASHKIFKMEKGKVV | 1 |
| H5-NA-3618-3662 | ACVNGSCFTVMTDGPSNGQASHKIFKMEKGKVVKSVELDAPNYHY | 4 |
| **H5-NA-3638-3662** | **HKIFKMEKGKVVKSVELDAPNYHY** | **8** |
| **H5-NA-3659-3689** | **NYHYEECSCYPNAGEITCVCRDNWHGSNRPW** | **9** |
| **H5-NA-3676-3854** | **CVCRDNWHGSNRPWVSFNQNLEYQIGYICSGVFGDNPRPNDGTGSCGPVSSNGAYGVKGFSFKYGNGVWIGRTKSTNSRSGFEMIWDPNGWTETDSSFSVKQDIVAITDWSGYSGSFVQHPELTGLDCIRPCFWVELIRGRPKESTIWTSGSSISFCGVNSDTVGWSWPDGAELPFTID** | **39** |
| H5-NA-3703-3723 | ICSGVFGDNPRPNDGTGSCGP | 2 |
| H5-NA-3704-3840 | CSGVFGDNPRPNDGTGSCGPVSSNGAYGVKGFSFKYGNGVWIGRTKSTNSRSGFEMIWDPNGWTETDSSFSVKQDIVAITDWSGYSGSFVQHPELTGLDCIRPCFWVELIRGRPKESTIWTSGSSISFCGVNSDTVG | 23 |
| H5-NA-3758-3809 | EMIWDPNGWTETDSSFSVKQDIVAITDWSGYSGSFVQHPELTGLDCIRPCFW | 2 |
| H5-NA-3821-3854 | TIWTSGSSISFCGVNSDTVGWSWPDGAELPFTID | 4 |
| **H5-NA-3834-3854** | **VNSDTVGWSWPDGAELPFTID** | **19** |
| **H5-M1-3859-3889** | **MSLLTEVETYVLSIIPSGPLKAEIAQKLEDV** | **65** |
| H5-M1-3859-4013 | MSLLTEVETYVLSIIPSGPLKAEIAQKLEDVFAGKNTDLEALMEWLKTRPILSPLTKGILGFVFTLTVPSERGLQRRRFVQNALNGNGDPNNMDRAVKLYKKLKREITFHGAKEVALSYSTGALASCMGLIYNRMGTVTTEVAFGLVCATCEQIA | 2 |
| H5-M1-3863-3909 | TEVETYVLSIIPSGPLKAEIAQKLEDVFAGKNTDLEALMEWLKTRPI | 35 |
| **H5-M1-3866-3894** | **ETYVLSIIPSGPLKAEIAQKLEDVFAGKN** | **102** |
| H5-M1-3989-4048 | IYNRMGTVTTEVAFGLVCATCEQIADSQHRSHRQMATITNPLIRHENRMVLASTTAKAME | 3 |
| H5-M1-4002-4110 | FGLVCATCEQIADSQHRSHRQMATITNPLIRHENRMVLASTTAKAMEQMAGSSEQAAEAMEIANQARQMVQAMRTIGTHPNSSAGLRDNLLENLQAYQKRMGVQMQRFK | 10 |
| H5-M1-4012-4055 | IADSQHRSHRQMATITNPLIRHENRMVLASTTAKAMEQMAGSSE | 5 |
| **H5-M1-4040-4104** | **ASTTAKAMEQMAGSSEQAAEAMEIANQARQMVQAMRTIGTHPNSSAGLRDNLLENLQAYQKRMGV** | **174** |
| H5-M1-4050-4078 | MAGSSEQAAEAMEIANQARQMVQAMRTIG | 2 |
| H5-M1-4054-4110 | SEQAAEAMEIANQARQMVQAMRTIGTHPNSSAGLRDNLLENLQAYQKRMGVQMQRFK | 88 |
| **H5-M1-4080-4109** | **HPNSSAGLRDNLLENLQAYQKRMGVQMQRF** | **45** |
| **H5-M2-4115-4123** | **SLLTEVETP** | **37** |
| **H5-M2-4124-4138** | **TRNEWECRCSDSSDP** | **29** |
| **H5-M2-4181-4209** | **VPESMREEYRQEQQSAVDVDDGHFVNIEL** | **17** |
| H5-NS1-4220-4284 | SSFQVDCFLWHVRKRFADQELGDAPFLDRLRRDQKSLRGRGNTLGLDIETATRAGKQIVERILEG | 10 |
| **H5-NS1-4236-4255** | **ADQELGDAPFLDRLRRDQKS** | **6** |
| H5-NS1-4283-4294 | EGESDKALKMPA | 1 |
| H5-NS1-4378-4397 | TGEDVKNAIGVLIGGLEWND | 1 |
| **H5-NS1-4393-4428** | **LEWNDNTVRVTETIQRFAWRNSDEDGRLPLPPNQKR** | **5** |
| **H5-NS2-4468-4509** | **SLKLYRDSLGETVMRMGDFHSLQIRNGKWREQLSQKFEEIRW** | **5** |
