# Supplementary material for: Antigenic Fingerprinting of H5N1 Avian Influenza Using Convalescent Sera and Monoclonal Antibodies Reveals Potential Vaccine and Diagnostic Targets
Source: PLoS Med. 2009 Apr 21;6(4):e1000049. doi: 10.1371/journal.pmed.1000049 (PMC2661249; doi:10.1371/journal.pmed.1000049)
Supplement: Table S2 — ELISA reactivity of sera from ten individuals with culture-confirmed seasonal influenza infections during the 2006–2007 seasons. End-point antibody titers (based on 5-fold dilutions starting at 1∶100) are reported for US 1–10 against the identical H5N1-Viet peptides used in Figures 4B and 6B). Student t-test was performed for each peptide reactivity using the end-point titers for the control sera (e.g., if end-point titer was <100, the value used for statistical analysis was 20), compared with the end-point titers of the five H5N1-convelescent samples (e.g., if end-point titer was >12,500, the value used for statistical analysis was 12,500). p-Values appear in the right column. (0.15 MB DOC) [file pmed.1000049.s006.doc]

| **SUPPLEMENTARY TABLE-2- END-POINT TITER OF CULTURE CONFIRMED SEASONAL INFLUENZA SERA FROM USA WITH H5N1 PEPTIDES** | | | | | | | | | | |  |
| --- | --- | --- | --- | --- | --- | --- | --- | --- | --- | --- | --- |
|  |  | | | | | | | | | |  |
| **PEPTIDE** | **IgG RESPONSES** | | | | | | | | | | ***T*-test** |
|  | **US-1** | **US-2** | **US-3** | **US-4** | **US-5** | **US-6** | **US-7** | **US-8** | **US-9** | **US-10** | ***p-value*** |
| **HA-2376-2659** | 500 | 100 | 100 | 500 | 100 | 100 | 100 | 100 | 500 | 500 | 4.38E-06 |
| **HA-2339-2581** | <100 | <100 | <100 | <100 | <100 | <100 | <100 | <100 | <100 | 100 | 3.34E-05 |
| **HA-2365-2427** | <100 | <100 | <100 | <100 | <100 | 100 | <100 | <100 | <100 | 100 | 0.005748 |
| **HA-2431-2453** | <100 | 100 | 100 | 100 | <100 | <100 | <100 | <100 | <100 | <100 | 0.037677 |
| **HA-2452-2481** | <100 | <100 | <100 | <100 | <100 | <100 | <100 | <100 | 100 | <100 | 8.6E-06 |
| **HA-2517-2538** | <100 | <100 | <100 | <100 | <100 | 100 | <100 | <100 | <100 | <100 | 8.6E-06 |
| **HA-2627-2669** | <100 | <100 | <100 | 100 | <100 | 100 | <100 | <100 | <100 | 100 | **0.06893** |
| **HA-2641-2685** | <100 | <100 | <100 | <100 | <100 | <100 | <100 | <100 | <100 | <100 | 3.62E-06 |
|  |  |  |  |  |  |  |  |  |  |  |  |
| **HA-2682-2703** | <100 | <100 | <100 | <100 | <100 | <100 | <100 | <100 | <100 | <100 | 0.003198 |
| **HA-2695-2756** | <100 | <100 | <100 | 100 | <100 | <100 | <100 | 100 | <100 | <100 | 0.018253 |
| **HA-2703-2731** | <100 | <100 | <100 | <100 | <100 | <100 | <100 | <100 | <100 | <100 | 3.97E-15 |
| **HA-2722-2762** | <100 | <100 | <100 | <100 | <100 | <100 | <100 | <100 | <100 | <100 | 3.97E-15 |
| **HA-2759-2814** | <100 | <100 | <100 | <100 | <100 | <100 | <100 | <100 | <100 | <100 | 5.5E-06 |
| **HA-2838-2866** | <100 | <100 | <100 | <100 | <100 | <100 | <100 | <100 | <100 | <100 | 0.000205 |
|  |  |  |  |  |  |  |  |  |  |  |  |
| **NA-3676-3854** | 100 | <100 | <100 | <100 | <100 | <100 | <100 | <100 | <100 | <100 | 0.000207 |
| **NA-3431-3481** | <100 | <100 | <100 | <100 | <100 | <100 | <100 | <100 | <100 | <100 | 0.016311 |
| **NA-3489-3530** | <100 | <100 | <100 | <100 | <100 | <100 | <100 | <100 | <100 | <100 | 5.5E-06 |
| **NA-3541-3576** | <100 | <100 | <100 | <100 | <100 | <100 | <100 | <100 | <100 | <100 | 0.000342 |
| **NA-3638-3662** | <100 | <100 | <100 | <100 | <100 | <100 | <100 | <100 | <100 | <100 | 0.011404 |
| **NA-3659-3689** | <100 | <100 | <100 | <100 | <100 | <100 | <100 | <100 | <100 | <100 | 0.016311 |
| **NA-3834-3854** | 100 | <100 | <100 | <100 | <100 | <100 | <100 | <100 | <100 | <100 | 0.016991 |
|  |  |  |  |  |  |  |  |  |  |  |  |

|  |  |  |  |  |  |  |  |  |  |  |  |
| --- | --- | --- | --- | --- | --- | --- | --- | --- | --- | --- | --- |
| **PEPTIDE** | **IgG RESPONSES** | | | | | | | | | | ***t*-test** |
|  | **US-1** | **US-2** | **US-3** | **US-4** | **US-5** | **US-6** | **US-7** | **US-8** | **US-9** | **US-10** | ***(p-value)*** |
|  |  |  |  |  |  |  |  |  |  |  |  |
| **PB2-344-375** | <100 | <100 | <100 | <100 | <100 | <100 | <100 | <100 | <100 | <100 | 0.000342 |
| **PB1-1348-1361** | <100 | <100 | <100 | <100 | <100 | <100 | <100 | <100 | <100 | <100 | 5.5E-06 |
| **PB1-1420-1437** | <100 | <100 | <100 | <100 | <100 | <100 | <100 | <100 | <100 | <100 | 5.5E-06 |
| **PB1-F2-1524-1598** | <100 | <100 | <100 | <100 | <100 | <100 | <100 | <100 | <100 | <100 | 5.5E-06 |
| **PB1-F2-1525-1572** | <100 | <100 | <100 | <100 | <100 | <100 | <100 | <100 | <100 | <100 | 0.000342 |
| **PB1-F2-1560-1592** | <100 | <100 | <100 | <100 | <100 | <100 | <100 | <100 | <100 | <100 | 0.000342 |
| **PB1-F2-1570-1605** | <100 | <100 | <100 | <100 | <100 | <100 | <100 | <100 | <100 | <100 | 0.000342 |
| **PA-2202-2251** | <100 | 500 | 500 | 100 | <100 | 100 | <100 | <100 | <100 | <100 | **0.272268** |
|  |  |  |  |  |  |  |  |  |  |  |  |
| **NP-2906-2929** | <100 | <100 | <100 | <100 | <100 | <100 | <100 | <100 | <100 | <100 | 0.003198 |
| **NP-2955-3011** | <100 | <100 | <100 | <100 | <100 | <100 | <100 | <100 | <100 | <100 | **0.075978** |
| **NP-3263-3305** | <100 | <100 | <100 | <100 | <100 | <100 | <100 | <100 | <100 | <100 | 0.006472 |
| **NP-3347-3384** | 100 | <100 | <100 | <100 | <100 | <100 | <100 | <100 | <100 | 100 | 0.00732 |
|  |  |  |  |  |  |  |  |  |  |  |  |
| **M1-3866-3894** | 500 | 500 | >12,500 | 500 | >12,500 | <100 | <100 | <100 | >12,500 | 2500 | **0.553319** |
| **M1-3859-3909** | 12,500 | 500 | >12,500 | 500 | >12,500 | <100 | <100 | 500 | >12,500 | 2500 | **0.837456** |
| **M1-4040-4104** | 12,500 | <100 | <100 | <100 | 2,500 | <100 | <100 | 500 | >12,500 | 100 | **0.780613** |
| **M1-4080-4109** | 5000 | 12,500 | <100 | <100 | 12,500 | <100 | <100 | <100 | 12,500 | 100 | **0.35878** |
|  |  |  |  |  |  |  |  |  |  |  |  |
| **M2e-4115-4138** | <100 | <100 | <100 | <100 | <100 | <100 | <100 | <100 | <100 | <100 | 0.005819 |
| **M2-4180-4209** | <100 | <100 | <100 | <100 | <100 | <100 | <100 | <100 | <100 | <100 | 0.003198 |
|  |  |  |  |  |  |  |  |  |  |  |  |
| **NS1-4236-4255** | 100 | <100 | <100 | <100 | 100 | <100 | <100 | <100 | <100 | <100 | 0.005748 |
| **NS1-4393-4428** | <100 | <100 | <100 | <100 | <100 | <100 | <100 | <100 | <100 | <100 | 5.5E-06 |
| **NS2-4468-4509** | <100 | <100 | <100 | <100 | <100 | <100 | <100 | <100 | <100 | <100 | 0.003198 |
